# Metabolic Recoding of NSUN2‐Mediated m^5^C Modification Promotes the Progression of Colorectal Cancer via the NSUN2/YBX1/m^5^C‐ENO1 Positive Feedback Loop

**DOI:** 10.1002/advs.202309840

**Published:** 2024-05-20

**Authors:** Baoxiang Chen, Yanrong Deng, Yuntian Hong, Lifang Fan, Xiang Zhai, Heng Hu, Siyuan Yin, Quanjiao Chen, Xiaoyu Xie, Xianghai Ren, Jianhong Zhao, Congqing Jiang

**Affiliations:** ^1^ Department of Colorectal and Anal Surgery Zhongnan Hospital of Wuhan University Wuhan 430071 China; ^2^ Hubei Key Laboratory of Intestinal and Colorectal Diseases Zhongnan Hospital of Wuhan University Wuhan 430071 China; ^3^ Clinical Center of Intestinal and Colorectal Diseases of Hubei Province Zhongnan Hospital of Wuhan University Wuhan 430071 China; ^4^ Department of Pathology Zhongnan Hospital of Wuhan University Wuhan 430071 China; ^5^ CAS Key Laboratory of Special Pathogens and Biosafety CAS Center for Influenza Research and Early Warning Wuhan Institute of Virology Chinese Academy of Sciences Wuhan 430064 China

**Keywords:** 5‐methylcytosine, colorectal cancer, ENO1, lactylation, NSUN2, YBX1

## Abstract

The RNA modification, 5‐methylcytosine (m^5^C), has recently gained prominence as a pivotal post‐transcriptional regulator of gene expression, intricately intertwined with various tumorigenic processes. However, the exact mechanisms governing m^5^C modifications during the onset and progression of colorectal cancer (CRC) remain unclear. Here, it is determined that the m^5^C methyltransferase NSUN2 exhibits significantly elevated expression and exerts an oncogenic function in CRC. Mechanistically, NSUN2 and YBX1 are identified as the “writer” and “reader” of ENO1, culminating in the reprogramming of the glucose metabolism and increased production of lactic acid in an m^5^C‐dependent manner. The accumulation of lactic acid derived from CRC cells, in turn, activates the transcription of NSUN2 through histone H3K18 lactylation (H3K18la), and induces the lactylation of NSUN2 at the Lys356 residue (K356), which is crucial for capturing target RNAs. Together, these findings reveal an intriguing positive feedback loop involving the NSUN2/YBX1/m^5^C‐ENO1 signaling axis, thereby bridging the connection between metabolic reprogramming and epigenetic remodeling, which may shed light on the therapeutic potential of combining an NSUN2 inhibitor with immunotherapy for CRC.

## Graphical abstract

The RNA modification, 5‐methylcytosine (m^5^C), has recently gained prominence in regulating gene expression and tumorigenesis. This study identifies NSUN2 and YBX1 as the m^5^C “writer” and “reader” of ENO1 in colorectal cancer, culminating in a positive feedback loop involving the NSUN2/YBX1/m^5^C‐ENO1 signaling axis, thereby bridging the connection between metabolic reprogramming and epigenetic remodeling.

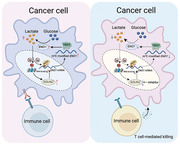

## 1 Introduction

Epigenetic modifications involved in the mitotically heritable and stable modifications that modulate gene expression without altering the underlying DNA sequences.^[^ ^1^ ^]^ Generally, epigenetic dysregulation in cancer manifests as mutations, abnormal expression of epigenetic modifying enzymes, and alterations in related cofactor levels.^[^ ^2^, ^3^ ^]^ These alterations drive changes in downstream gene expression through processes such as chromatin remodeling, DNA methylation, RNA methylation, and histone modification, all of which contribute to cancer initiation and progression.^[^ ^2^, ^3^, ^4^, ^5^ ^]^ RNA modifications, notably N6‐methyladenosine (m^6^A), govern a broad spectrum of cellular functions in both physiological and pathological contexts.^[^ ^5^, ^6^, ^7^ ^]^ Of these, 5‐methylcytosine (m^5^C) represents another prevalent RNA modification involved in RNA metabolic regulation and various tumorigenic processes.^[^ ^8^, ^9^, ^10^, ^11^, ^12^, ^13^, ^14^ ^]^ Colorectal cancer (CRC) ranks as the second leading cause of cancer‐related fatalities and stands as the third most prevalent malignant tumor on a global scale.^[^ ^15^, ^16^ ^]^ Although the primary treatment options include surgery, chemotherapy, and radiotherapy, the prognosis for CRC patients remains poor, marked by a 5‐year survival rate of only 13.1% in cases of metastatic CRC.^[^ ^16^ ^]^ Gaining insights into the evolutionary and metastatic mechanisms of CRC is crucial for the development of precise and effective treatments. Accumulating studies have substantiated the intricate association of m^5^C modification with the carcinogenesis and tumorigenesis of various human cancers, including bladder cancer (BLCA), esophageal carcinoma (ESCA), stomach adenocarcinoma (STAD), and liver hepatocellular carcinoma (LIHC).^[^ ^10^, ^13^, ^17^, ^18^, ^19^ ^]^ Nonetheless, the full elucidation of whether m^5^C modifications potentially contribute to driving the development of CRC is still pending.

Metabolic reprogramming stands out as one of the hallmark features of cancer cells, characterized by disruptions in glucose metabolism, the tricarboxylic acid cycle, fatty acid metabolism, amino acid metabolism, and nucleotide synthesis.^[^ ^20^, ^21^ ^]^ Mutations in oncogenes/tumor suppressor genes trigger the reconfiguration of metabolism and epigenetic modifications while being regulated by epigenetic modifications and alterations in metabolite levels.^[^ ^22^, ^23^ ^]^ Distinct metabolic alterations in cancer, combined with factors in the tumor microenvironment (TME) and dietary interventions, can actively engage in epigenetic regulation by modifying the activity or substrate abundance of epigenetic modification enzymes and cofactors through shifts in metabolite levels.^[^ ^22^ ^]^ In contrast, alterations in the expression or activity of epigenetical regulators can elicit a broad spectrum of direct and indirect effects on cellular metabolism in cancer.^[^ ^22^ ^]^ In the context of the TME, the substantial amount of lactic acid stemming from glycolysis, previously considered the metabolic byproduct or waste product, is now recognized as a key factor being involved in cancer cell metastasis, angiogenesis, therapeutic resistance, and immune evasion.^[^ ^24^, ^25^, ^26^, ^27^ ^]^ Moreover, recent studies have unveiled that lactic acid can serve as a substrate for the modification of histone lysine residues, resulting in the emergence of a novel form of epigenetic modification called histone lactylation, whose modification directly stimulates gene transcription from the chromatin.^[^ ^28^, ^29^, ^30^, ^31^, ^32^, ^33^ ^]^ However, mechanisms underlying interactions between oncogene and tumor suppressor gene alterations, metabolic reprogramming, and epigenetic modification in cancer, as well as the full extent of their profound impact on tumorigenesis through abnormal crosstalk remain unknown.

Therefore, in this study, we determined the elevated expression levels of the m^5^C methyltransferase NSUN2 and its oncogenic role in CRC, as well as underlying genetic and molecular mechanisms. NSUN2‐induced metabolic reprogramming enhanced glucose metabolism by modulating the expression of ENO1 in an m^5^C‐dependent manner, resulting in the increased production of lactic acid in CRC cells. Also, the lactic acid not only activates the transcription of NSUN2 through histone H3K18 lactylation (H3K18la) but also induces the lactylation of NSUN2 at the Lys356 residue (K356), which is crucial for capturing target RNAs and m^5^C modification of *ENO1* mRNA. Moreover, an effective small‐molecule inhibitor of NSUN2 has been indentified in diminishing the enzymatic function and the expression of downstream targets, offering a novel and promising therapeutic option in the realm of cancer immunotherapy for CRC. Taken together, we uncovered an intriguing positive feedback loop that encompasses the NSUN2/YBX1/m^5^C‐ENO1 signaling axis, thereby bridging the connection between metabolic reprogramming and epigenetic remodeling.

## 2 Results

### 2.1 Elevated m^5^C Levels and Increased NSUN2 Expression in CRC

**Figure 1:**
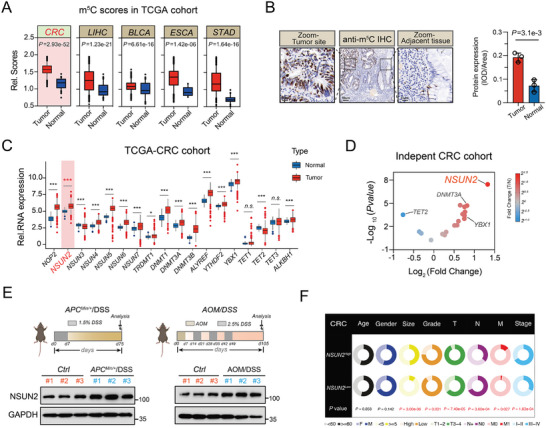
m^5^C scores and NSUN2 were found to be upregulated and associated with the progression and metastasis of CRC. A) Comparison of the relative m^5^C scores between cancer and normal tissues from the TCGA database. B) Representative immunohistochemical staining (left) and quantitative analysis (right) of m^5^C in the CRC patients. C) Comparison of the relative expression of 18 m^5^C regulatory genes between cancer and normal tissues from TCGA database. D) Rank‐ordered analysis of the fold change and *P*‐value for 18 m^5^C regulatory genes in the independent cohort of paired CRC patients. E) Expression of NSUN2 protein in control colon tissues (*Ctrl*) and tumor tissues from AOM/DSS‐inducted CRC mice or *APC^Min/+^/*DSS‐induced CRC mice (*APC^Min^* ^/+^/DSS). F) Pie charts visually representing the associations between clinicopathologic factors and NSUN2 protein expression in the CRC cohort. Data are presented as means ± SD; *P < 0.05, **P < 0.01, ***P < 0.001, n.s. non‐significant.

Accumulating studies have shown that m^5^C regulators play pivotal roles in the initiation and progression of a wide range of cancers.^[^ ^10^, ^13^, ^17^, ^18^, ^19^ ^]^ In this study, the m^5^C score was established using 18 m^5^C regulators identified in our previous publication.^[^ ^34^ ^]^ As expected, the m^5^C scores were significantly elevated in cancer tissues compared with those of normal tissues from TCGA, including CRC, LIHC, BLCA, ESCA, and STAD (**Figure** **1**A). Of these, the m^5^C modification has recently been confirmed to function as a carcinogenic process in LIHC, BLCA, ESCA, and STAD,^[^ ^10^, ^13^, ^17^, ^18^, ^19^ ^]^ prompting us to explore the potential tumorigenic role of m^5^C regulators in CRC. Similarly, immunohistochemistry (IHC) results from the CRC tissues demonstrated an elevated level of m^5^C modification at the tumor tissues in comparison to that of adjacent normal tissues (Figure 1B). In line with our previously published study, most (16/18) m^5^C regulatory genes were significantly differentially expressed between CRC and adjacent normal tissues from TCGA database (Figure 1C). Thus, the expression patterns of these regulatory genes were further validated in an independent cohort of patients with CRC. Strikingly, the m^5^C methyltransferase NSUN2 presented the most remarkable expression difference among CRC and paired paracancerous tissues, and was thus selected for further research (Figure 1D). Likewise, substantially elevated expression levels of the NSUN2 protein were observed in tumor tissues of azoxymethane/dextran sulfate sodium (AOM/DSS)‐ and DSS (*APC* ^Min/+^/DSS)‐induced CRC mouse models compared with those of normal colorectal tissues (Figure 1E). Additionally, in the independent paired CRC tissues from patients, NSUN2 protein levels were notably elevated in cancerous tissues compared to adjacent normal tissues. (Figure S1A,B, Supporting Information). Taken together, these results affirmed that NSUN2 exhibited high expression at both the transcriptional and protein levels in CRC. Subsequently, we extended our evaluation of NSUN2 expression to an independent cohort containing 126 CRC tissues using quantitative reverse transcription polymerase chain reaction (RT‐qPCR, Tables S1 and S2, Supporting Information). The clinicopathological correlation analysis revealed that the NSUN2 expression level was significantly associated with the tumor size and TNM stage of CRC (Figure 1F). Overall, these results demonstrated that NSUN2 may play a pro‐oncogenic role and metastasis‐promoting function in CRC, although the specific mechanism remains unclear.

### 2.2 NSUN2 Depletion Inhibits Tumorigenesis and Metastasis of CRC

**Figure 2:**
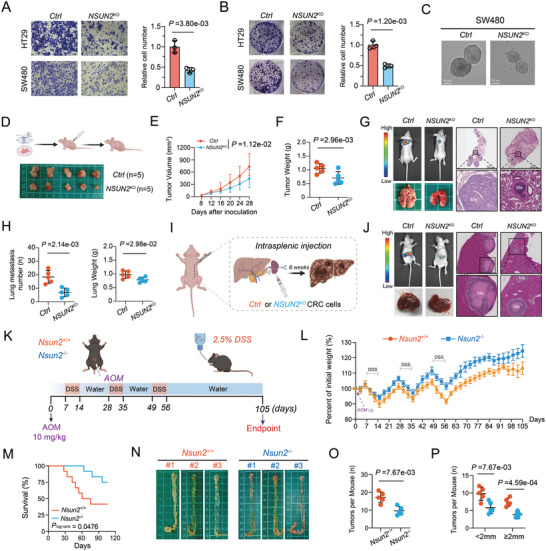
NSUN2‐deficency impedes the tumorigenesis and metastasis of CRC in vitro and in vivo. A) Representative images of the transwell assay in SW480 and HT29 cells (left), and the accompanying bar graphs display the relative invasive number of HT29 cell (right). B) Representative colony formation results of HT29 and SW480 cells with *NSUN2* knockout or control (left), and the accompanying bar graphs display the relative colony numbers of HT29 cell (right). C) Representative images of tumor sphere‐forming assay of CRC cells with *NSUN2* knockout or control. D) Representative picture showing subcutaneous tumor of *NSUN2* knockout and control groups in nude mice. E) Growth curves of subcutaneous tumors formed by control or *NSUN2* knockout of  CRC cells in nude mice. F) The weight of subcutaneous tumors formed in nude mice between the control or *NSUN2* knockout groups. G) Representative bioluminescence and HE staining images of lung metastasis in mice after tail vein injection of control or *NSUN2* knockout CRC cells. H) Statistical analysis of lung metastasis number and lung weight in control or *NSUN2* knockout groups. I) Schematic representation of the intra‐splenic liver metastasis mouse model of CRC. J) Representative bioluminescence and HE staining images of liver metastasis in mice after distal spleen injection of control or *NSUN2* knockout CRC cells. K) Schematic representation of the AOM/DSS induced‐orthotopic CRC mouse model. L) The body weight curves of *Nsun2* ^+/+^ and *Nsun2* ^−/−^ mice in AOM/DSS‐induced orthotopic CRC model. M) The survival curve of *Nsun2* ^+/+^ and *Nsun2* ^−/−^ mice in AOM/DSS‐induced orthotopic CRC model. N) Typical images of colon tumors from *Nsun2* ^+/+^ and *Nsun2* ^−/−^ mice in AOM/DSS‐induced orthotopic CRC model. O) Comparision of the total of colorectal tumor numbers in mice from *Nsun2* ^+/+^ and *Nsun2* ^−/‐^ groups. P) Comparision of the indicated size of colorectal tumor numbers in mice from *Nsun2* ^+/+^ and *Nsun2* ^−/‐^ groups.

To further determine whether NSUN2  functions as a tumor‐promoting factor in CRC, we constructed two *NSUN2*‐knockout (*NSUN2* ^KO^ or *NSUN2* ^−/−^) CRC cell lines using the CRISPR/ Cas9 gene editing system. Functionally, the depletion of NSUN2 significantly inhibited the proliferation and invasion ability of CRC cells (SW480 and HT29) (**Figure** **2**A,B; Figure S2A,B, Supporting Information). In addition, the tumor formation assay revealed that knockout of *NSUN2* remarkably suppressed sphere formation and cancer stemness in CRC cells (Figure 2C). Based on the above bioinformatic analysis and in vitro cell results, four mouse models were established to further verify the oncogenic function of NSUN2 in vivo: subcutaneous tumor formation, lung metastasis of CRC, liver metastasis of CRC, and AOM/DSS‐induced CRC mouse models. As expected, nude mice injected with *NSUN2* ^KO^ CRC cells showed significantly reduced subcutaneous tumor volume and weight, and exhibited fewer and smaller lung and liver metastases than the control group (Figure 2D–J). To better elucidate the oncogenic role of NSUN2 in CRC, *Nsun2* knockout (*Nsun2* ^−/−^; Figure S2C,D and Table S3, Supporting Information) mouse were generated and used to establish an orthotopic CRC model. After administration of AOM/DSS, *Nsun2^−/−^* mice developed fewer and smaller tumors with histological dysplasia than wild‐type (*Nsun2^+/+^*) mice (Figure 2K–P). Moreover, *Nsun2^−/‐^* mice had better prognoses than *Nsun2^+/+^* mice according to the survival analysis (Figure 2M). In summary, these results collectively validate the critical role of NSUN2 in CRC tumorigenesis and metastasis.

### 2.3 NSUN2‐Mediated m^5^C Modification Leads to the Reprogramming of Glucose Metabolism by Targeting ENO1 in CRC

**Figure 3:**
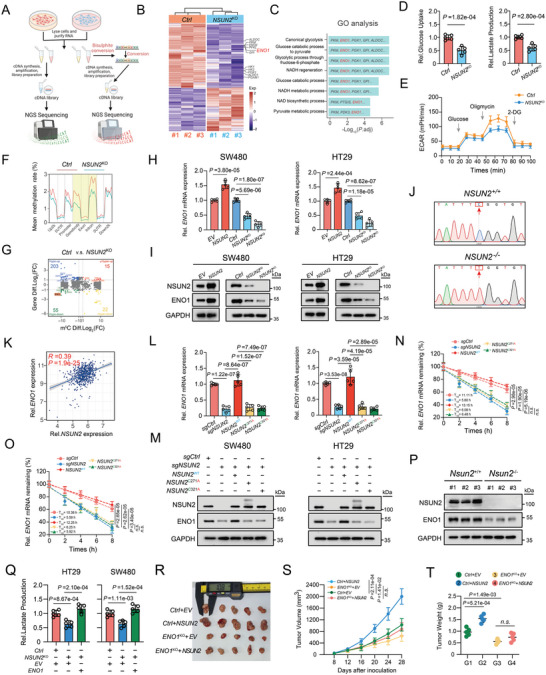
NSUN2‐mediated m^5^C modification leads to the reprogramming of glucose metabolism by targeting ENO1 in CRC. A) Illustration depicting the process of RNA‐Seq and RNA‐BiS‐Seq in CRC cells. B) Heatmap displaying the disparities in gene expression between the control and *NSUN2* knockout groups. C) GO analysis of the genes with differential expression between the control and *NSUN2* knockout CRC cell groups. D) Comparison of the relative glucose uptake and lactate production between control and *NSUN2* knockout SW480 cells. E) Analysis of ECAR in control and *NSUN2* knockout SW480 cells. F) Comparison of the mean methylation levels in various genomic regions between the control and *NSUN2* knockout groups. G) The distribution of mRNAs exhibiting significant alterations in both gene expression and m^5^C methylation levels, as determined through the integrated analysis of RNA‐Seq and RNA‐BiS‐Seq. H,I) Analyzing the mRNA and protein expression levels of ENO1 in CRC cells after treatment with the indicated vectors. J) Sanger‐based confirmation of m^5^C sites within *ENO1* RNA in control and *NSUN2* knockout CRC cells. K) The correlation analysis of the relative mRNA expression levels between *ENO1* and *NSUN2* in TCGA‐CRC database. L,M) The relative mRNA and protein expression levels of ENO1 in CRC cells after treatment with the indicated vectors. N‐O) Analysis of *ENO1* mRNA half‐life in SW480 and HT29 cells following treatment with the indicated vectors. P) The protein expression levels of NSUN2 and ENO1 in tumor tissues from *Nsun2* ^+/+^ and *Nsun2* ^−/‐^ mice with AOM/DSS‐induced CRC model. Q) The rescue experiment of lactate production subsequent to the overexpression of ENO1 in *NSUN2*‐knockout CRC cells. R) Images of the dissected subcutaneous tumors from the tumor‐bearing mice of in vivo rescue model (n = 5 mice per group). S) Time‐course evaluation of subcutaneous tumor volumes measured every 4 days in tumor‐bearing mice. T) The final weight of the subcutaneous tumors was shown in the scatter plot for indicated groups.

Given that NSUN2 is an important m^5^C methyltransferase, we explored how NSUN2 involved in the occurrence and progression of CRC in an m^5^C‐dependent manner. RNA sequencing (RNA‐Seq) and RNA bisulfite sequencing (RNA‐BiS‐Seq) were performed on *NSUN2* ^KO^ and control CRC cells (**Figure** **3**A). In total, 829 differentially expressed genes (DEGs) were identified using RNA‐Seq, including 482 downregulated genes and 347 upregulated genes (Figure 3B). The Gene ontology (GO) analysis revealed that the DEGs were enriched in glucose metabolism‐related pathways, including canonical glycolysis, the glucose catabolic process to pyruvate, the glycolytic process through fructose‐6‐phosphate, NADH regeneration, and glucose catabolic processes (Figure 3C). Consistently, the Kyoto Encyclopedia of Genes and Genomes (KEGG) analysis revealed that absence of NSUN2 affected carbon metabolism (central carbon metabolism) and glycolysis in CRC cells. (Figure S2A, Supporting Information). In line with these findings, *NSUN2* knockout decreased glucose consumption and lactate production, concomitantly suppressing the extracellular acidification rate (ECAR) in SW480 and HT29 cells (Figure 3D,E; Figure S2B–E, Supporting Information). Following that, we aimed to investigate how NSUN2 deletion dampened glycolytic metabolism in CRC cells. As anticipated, BiS‐Seq analysis revealed a significant genome‐wide reduction in methylation levels at different chromosomes and gene regions in the *NSUN2* knockout group (Figure 3F; Figure S3E–G, Supporting Information). The comprehensive analysis of RNA‐BiS‐Seq and RNA‐Seq datas demonstrated that both the m^5^C methylation and mRNA levels of *ENO1* were significantly decreased in *NSUN2* knockout CRC cells compared with those in control group (Figure 3G). Accordingly, we examined the effect of NSUN2‐mediated m^5^C modification on ENO1. RT‐qPCR analysis and western blotting indicated that *NSUN2* knockdown and *NSUN2* knockout significantly reduced *ENO1* mRNA (Figure 3H) and protein levels (Figure 3I) in CRC cells, whereas *NSUN2* overexpression yielded opposing results (Figure 3H,I). Bisulfite‐PCR sequencing with ENO1‐specific primers revealed total elimination of methylation at the *ENO1* (chr1:C8878634) site in NSUN2‐deficient CRC cells (Figure 3J and Table S4, Supporting Information). In TCGA data analysis, the RNA expression of *NSUN2* exhibited a significantly positive association with that of *ENO1* level in CRC (Figure 3K). To further validate whether the oncogenic function of NSUN2 in regulating ENO1 depends on m^5^C methyltransferase activity, two enzymatically non‐functional mutants of NSUN2 were created by introducing point mutations at the release site (cysteine 271) or the catalytic site (cysteine 321), with the aim of disrupting its m^5^C enzymatic activity.^[^ ^35^ ^]^ Indeed, overexpression of the wild‐type (WT) of NSUN2, but not mutant NSUN2 (*NSUN2* ^C271A^ or *NSUN2* ^C321A^), led to the restoration of *ENO1* mRNA (Figure 3L) and protein levels (Figure 3M) in *NSUN2* ^KO^ CRC cells. It is plausible that NSUN2‐mediated m^5^C modification sustains the expression of *ENO1* by augmenting mRNA stability. As shown in Figure 3N,O, the half‐life of *ENO1* RNA was markedly decreased in *NSUN2* ^KO^ cells following actinomycin D treatment, whereas this reduction was reversed upon overexpression of the *NSUN2* ^WT^, but not the *NSUN2* ^C271A^ or *NSUN2* ^C321A^ mutant. Consistently, a higher level of ENO1 was also observed in the histologic dysplasia of AOM/DSS‐induced *Nsun2* ^+/+^ mice than that in *Nsun2* ^−/−^ mice (Figure 3P). In the in vitro rescue experiment, the overexpression of ENO1 reversed the impaired proliferation and invasion abilities, along with the glucose metabolism, in *NSUN2*‐knockout CRC cells (Figure 3Q; Figure S3H–J, Supporting Information). Yet, in the in vivo experiment, the overexpression of NSUN2 in the context of *ENO1* knockout failed to effectively resume the reduced subcutaneous implantation of CRC tumors, suggesting that ENO1 may acts as a critical downstream target of NSUN2 (Figure 3R–T). All of these findings demonstrate the pivotal role of ENO1 in NSUN2‐mediated m^5^C modification, contributing to the reprogramming of glucose metabolism in CRC.

### 2.4 YBX1 Functions as an m^5^C “Reader” to Recognize and Stabilize ENO1

**Figure 4:**
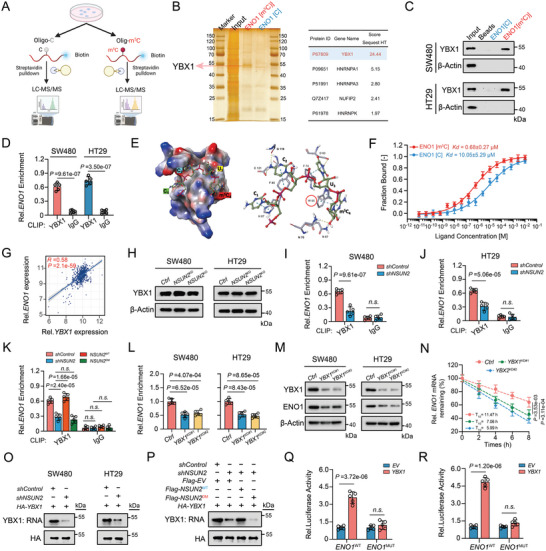
YBX1 acts as an m^5^C “reader”, identifying and stabilizing *ENO1* mRNA. A) Illustration depicting the process of RNA‐pulldown and mass spectrometry conducted in CRC cells. B) Analysis of proteins in the Input and RNA‐pulldown groups was conducted using SDS/PAGE followed by silver staining. C) Western blotting analysis of potential ENO1[m^5^C] binding proteins obtained from RNA pulldown followed by mass spectrometry analysis. D) PAR‐CLIP‐qPCR analysis of binding ability of YBX1 to ENO1 in CRC cells. E) Electrostatic potential of the surface of YBX1 CSD in complex with the *ENO1* m^5^C RNA oligo. Recognition of m^5^C by the YBX1 CSD domain. F) Quantification of the binding affinity of the unmodified or m^5^C‐modified *ENO1* RNA oligomers to the YBX1 CSD by MST. G) The correlation analysis of the relative mRNA expression levels between *ENO1* and *YBX1* in TCGA‐CRC database. H) Western blotting analysis of YBX1 protein level in CRC cells with *NSUN2* knockdown or knockout. I) PAR‐CLIP‐qPCR analysis of binding ability of YBX1 to *ENO1* RNA in SW480 cell with *NSUN2* knockdown. J) PAR‐CLIP‐qPCR analysis of binding ability of YBX1 to *ENO1* RNA in HT29 cell with *NSUN2* knockdown. K) PAR‐CLIP‐qPCR analysis of binding ability of YBX1 to *ENO1* RNA in SW480 cell with indicated vectors. L) RT‐qPCR analysis of the relative *ENO1* mRNA expression level in CRC cells with *YBX1* knockdown. M) Western blotting analysis of the relative ENO1 protein expression level in CRC cells with *YBX1* knockdown. N) The RNA half‐life analysis of *ENO1* mRNA in SW480 cell with *YBX1* knockdown. O) PAR‐CLIP assay of RNA pulled down by HA‐YBX1 in *NSUN2*‐knockdown SW480 and HT29 cells. P) Rescue PAR‐CLIP assay of RNA pulled down by HA‐YBX1 in *NSUN2*‐knockdown SW480 cells transfected with *Flag‐EV*, *Flag‐NSUN2* ^WT^ or *Flag‐NSUN2* ^DM^. Q) Luciferase reporter assays of luciferase reporter gene containing the wild‐type ENO1‐m^5^C site (*ENO1* ^WT^) or the mutant m^5^C site (*ENO1* ^MUT^) in both control or stable *YBX1* overexpression SW480 cells. R) Luciferase reporter assays of luciferase reporter gene containing the *ENO1* ^WT^ or the *ENO1* ^MUT^ in both control or stable YBX1 overexpression HT29 cells.

It's noteworthy that m^5^C‐methylated RNAs are recognized and stabilized by specific reader proteins.^[^ ^10^, ^18^ ^]^ To identify the potential m^5^C methylation reader of *ENO1* RNA, mass spectrometry analysis was conducted using proteins pulled down from the biotin‐labeled *ENO1* RNA or biotin‐labeled m^5^C‐*ENO1* RNA probes (**Figure** **4**A; Table S5, Supporting Information). In the biotin‐labeled m^5^C‐*ENO1* RNA group, five proteins were detected, with YBX1 presenting the highest score, a finding also corroborated by RNA‐pulldown assay (Figure 4B,C; Figure S4A, Supporting Information). The results of the photoactivatable‐ribonucleoside‐enhanced crosslinking and immunoprecipitation (PAR‐CLIP) analysis further confirmed the direct binding of YBX1 to m^5^C‐*ENO1* in both SW480 and HT29 cells (Figure 4D). Because YBX1 possesses a conserved cold shock domain (CSD),^[^ ^10^, ^18^, ^36^ ^]^ which has been shown to target m^5^C‐modified RNAs,^[^ ^10^, ^37^ ^]^ we hypothesized that m^5^C‐modified *ENO1* binds to YBX1 through this domain. To further identify the crucial residues of YBX1 involved in binding to the m^5^C site of ENO1, structural modeling was conducted for the complex of YBX1 with the m^5^C‐containing *ENO1* RNA hexamer oligo. Based on the modeled result of YBX1‐m^5^C *ENO1* RNA complex structure, H87, F85, F74, and W65 appear to be the crucial interacting residues, and N67 and D70 had direct hydrogen bonds with the m5C RNA fragment (Figure 4E; Figure S4B,C Supporting Information). The microscale thermophoresis (MST) assay revealed that the YBX1 binds to m^5^C‐modified RNA oligo with a dissociation constant (KD) of 0.68 ± 0.27 µM, whereas the binding affinity for unmethylated RNA oligo was notably lower (KD = 10.05 ± 5.29 µM; Figure 4F), signifying that the YBX1's perferential binding to m^5^C‐modified RNA oligos. In TCGA database, the RNA expression of *NSUN2* exhibited a significantly positive association with that of *ENO1* level in CRC (Figure 4G). The YBX1 expression was unaltered in *NSUN2* knockdown or knockout CRC cells (Figure 4H). YBX1‐PAR‐CLIP further indicated that the direct binding interaction of YBX1 to *ENO1* were significanlty reduced in *NSUN2* knockdown CRC cells (Figure 4I,J). More specifically, this effect was prominently reversed by the *NSUN2* ^WT^, while the m^5^C‐methyltransferase‐defective NSUN2 (double mutant NSUN2, *NSUN2* ^DM^) failed to produce the same effect (Figure 4K). Given the ability of YBX1 to bind to and stabilize the target m^5^C‐modified RNA, we assume that the enhanced stability of *ENO1* RNA by NSUN2 depends, at least in part, on YBX1. Moreover, the RNA and protein expression levels of ENO1 were significantly reduced following YBX1 knockdown (Figure 4L,M). Similarly, the half‐life of *ENO1* RNA was markedly decreased in YBX1‐knockdown cells following actinomycin D treatment (Figure 4N; Figure S4D, Supporting Information). The YBX1 RNA‐binding affinity, regulated by NSUN2, was further evaluated, and substantial decreases were observed in SW480 and HT29 cells following *NSUN2* knockdown (Figure 4O), whereas overexpressed *NSUN2* ^WT^ not double mutant NSUN2 rescued this effect (Figure 4P). Ultimately, the luciferase activity of *ENO1* ^WT^ was markedly increased in YBX1 overexpression cells, with no such effect observed for *ENO1* ^MUT^ (Figure 4Q,R). Collectively, these results indicate that YBX1 serves as an classical reader, recognizing and stabilizing the m^5^C‐modified *ENO1* mRNA.

### 2.5 Tumor‐Derived Lactic Acid Induced the Upregulation of NSUN2 Expression via Histone Lactylation

**Figure 5:**
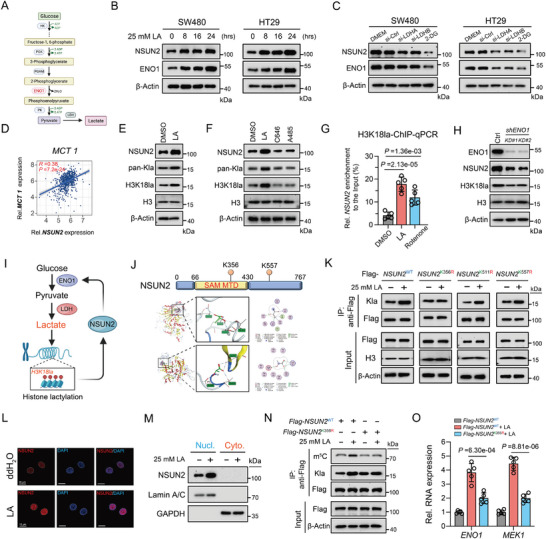
Tumor‐derived lactic acid activates the transcription of *NSUN2* through histone H3K18la, and directly induces the lactylation of NSUN2 at the K356. A) Metabolic flowchart illustrating the functional role of ENO1 in glucose metabolism.B) Western blotting analysis of the indicated proteins from the whole‐cell lysate of CRC cells treated with 25 mm L‐lactic acid at indicated time. C) Western blotting analysis of NSUN2 and ENO1 protein levels of CRC cells treated with si‐NC or si‐LDHA or si‐LDHB or 2‐DG. D) The correlation analysis of the relative mRNA expression levels between *NSUN2* and *MCT1* in TCGA‐CRC database. E) Western blotting analysis of the indicated protein levels from the whole‐cell lysate of CRC cells with addition of 25 mm L‐lactic acid. F) Western blotting analysis of the indicated proteins from the whole‐cell lysate of CRC cells treated with 25 mm L‐lactic acid, or histone acetyltransferase p300 inhibitors (C646 or A485) for 24h. G) ChIP‐qPCR analysis of the relative NSUN2 level to the Input in CRC cells treated with 25 mm L‐lactic acid or 500 nm rotenone for 24 h. H) Western blotting analysis of the indicated proteins from the whole‐cell lysate of CRC cells with *ENO1* knockdown. I) The diagram illustrates NSUN2 promoting the transcription of NSUN2 through ENO1 regulation, forming a positive feedback loop. J) Molecular docking for prediction of the binding affinity between the NSUN2 and L‐lactic acid molecule. L) Western blotting analysis of the relative expression of NSUN2 in nuclear and cytoplasmic fractions in CRC cells treated with or without 25 mm L‐lactic acid for 24 h. M) Immunofluorescence analysis of NSUN2 protein in SW480 cell treated with or without 25 mm L‐lactic acid for 24 h. N) Immunoprecipitation and western blotting analysis of indicated targets in CRC cells treated with indicated vectors and L‐lactic acid after UV‐crosslinked. O) RT‐qPCR analysis of the relative RNA expression levels in CRC cells with different treatment.

Our above results show that NSUN2 is abnormally high expressed in CRC, but the detailed mechanism remains unclear. ENO1, as the classical enzyme, plays a crucial role in glucose metabolism and cancer development (**Figure** **5**A), while high expression of NSUN2 leads to the reprogramming of glucose metabolism via m^5^C modification of ENO1 in CRC. Hence, we investigated whether the end product of glucose metabolism, lactate, was responsible for the upregulation of NSUN2 expression. Western blot analysis demonstrated a progressive rise in NSUN2 protein expression in CRC cells over time following the addition of lactic acid (Figure 5B). Lactate dehydrogenases (LDH) are key metabolic enzymes involved in glycolysis that contribute to lactic acid production.^[^ ^38^ ^]^ The small interfering RNAs (siRNAs) for LDHA and LDHB, and 2‐deoxyglucose (2‐DG, an inhibitor of glucose metabolism) were employed to mitigate lactate production, and the expression of both NSUN2 and ENO1 protein were reduced with these treaments (Figure 5C). Moreover, monocarboxylate transporters (MCTs) transport lactate across the cell membrane.^[^ ^39^ ^]^ Remarkably, the RNA expression levels of *MCT1* and *MCT3* were positively correlated with that of *NSUN2*, as evident from TCGA database (Figure 5D; Figure S5A, Supporting Information). Previous studies indicated that histone lactylation directly stimulates chromatin gene transcription; thus, we then attempted to investigate whether histone lactylation is responsible for facilitating NSUN2 expression. After the addition of lactic acid, the expression levels of both NSUN2 and Pan‐Kla were elevated, accompanied by a notable increasion in the H3K18la level, whereas no significant changes were observed in the levels of H3K23la, H4K8la, and H4K12la (Figure 5E; Figure S5B, Supporting Information). Acetyltransferase p300 has been recognized as a potential “writer” enzyme for histone Kla^[^ ^30^ ^]^; in this study, we found that pan‐Kla, H3K18la, and NSUN2 expression levels were decreased following treatment with the two p300 inhibitors (A485 and C646) (Figure 5F). Chromatin immunoprecipitation (ChIP) with an anti‐H3K18la antibody followed by qPCR was then conducted to elucidate the regulatory role of histone lactylation in NSUN2 expression, revealing the enrichment of H3K18la in the promoter regions of NSUN2 (Figure 5G). Given that histone lactylation is dependent on the presence of a substrate produced by glycolysis and considering our finding that NSUN2‐mediated m^5^C modification leads to the reprogramming of glucose metabolism by targeting ENO1, it seems plausible that an interesting positive feedback loop might exist between histone H3K18la and m^5^C‐mediated regulation of glycolysis that tandems the epigenetic switch/metabolic reprogramming in CRC progression. We confirmed that the targeted reduction in ENO1 expression also decreased the expression of NSUN2 and H3K18la levels, further validating the existence of this positive feedback regulatory loop (Figure 5H,I).

### 2.6 Lactylation of NSUN2 Enhanced the Capture of m^5^C‐Modified RNA via the MTD

Multiple essential post‐translational modifications (PTMs) have been detected in NSUN2.^[^ ^17^ ^]^ Of these, SUMOylation is known to suppress methyltransferase activity.^[^ ^17^ ^]^ Metabolites possess the potential to induce protein‐specific PTMs; therefore, we explored novel modes of lactic acid modification that may regulate the function of NSUN2, such as investigating whether direct lactylation of NSUN2 is involved. A comprehensive pan‐cancer proteomic analysis revealed a potential lactylated amino acid site on NSUN2.^[^ ^40^ ^]^ In addition, molecular docking in the Molecular Operating Environment (MOE) was employed to predict the binding affinity between L‐lactic acid molecules and NSUN2 protein, and the results indicated that two lysine sites were predicted to be potentially lactylated (Figure 5J). Moreover, to prevent indirect modifications, we disrupted protein‐protein interactions with sodium dodecyl sulfate (SDS), followed by immunoprecipitation (IP) and western blot analysis, and the results indeed confirmed the presence of Kla on NSUN2 (Figure 5K). Thus, we mutated these three potential lactylation sites by substituting lysine (K) with arginine (R). Western blotting after anti‐Flag immunoprecipitation confirmed direct lactylation of the K356 site within the NSUN2 protein (Figure 5K). In an effort to investigate the impact of the lactylation of NSUN2 on its nuclear localization, immunofluorescence and protein nucleocytoplasmic separation were performed in CRC cells following the addition of lactic acid, and the results consistently showed no alternation in subcellular localization following lactic acid treatment (Figure 5L‐M). It is however, worth noting that K356 was situated in the MTD (Figure 5J), in close proximity to the catalytic site of NSUN2. Accordingly, we investigated whether direct lactylation of NSUN2 affects its ability to bind to m^5^C‐modified RNA. *Flag‐NSUN2* ^WT^ or *Flag‐NSUN2* ^K356R^ were transfected into CRC cells, and UV crosslinking was subsequently performed. Then, the UV‐crosslinked cells were lysed for IP with an anti‐Flag antibody, followed by western blotting with anti‐m^5^C antibody, and the results indicated that the m^5^C‐modified RNAs bound by *Flag‐NSUN2* ^WT^ were more abundant than those bound by *Flag‐* *NSUN2* ^K356R^ (Figure 5N). To gain deeper insights into the function of NSUN2 lactylation, we transfected the *Flag‐* *NSUN2* ^WT^ or *Flag‐* *NSUN2* ^K356R^ variant into CRC cells without or with lactic acid treatment. Significantly, the expression of several m^5^C modification‐mediated genes were increased in CRC cells transfected with the *NSUN2* ^WT^ compared with that of cells transfected with the *NSUN2* ^K356R^ in the lactic acid treatment group (Figure 5O; Figure S5C, Supporting Information). In summary, our data conclusively demonstrated that tumor‐derived lactic acid facilitates the direct lactylation of NSUN2, enhancing its RNA‐binding capacity and thereby contributing to the m^5^C‐mediated progression and metastasis of CRC.

### 2.7 Identification of an Efficacious Small‐Molecule Inhibitor of NSUN2 in Combination with Immunotherapy

**Figure 6:**
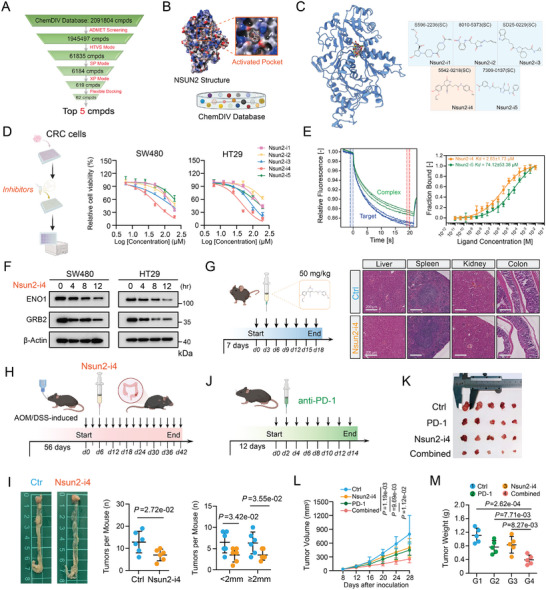
Identification of an efficacious small‐molecule inhibitor of NSUN2 in combination with immunotherapy. A) Pyramid flowchart of the pipeline to identify NSUN2 inhibitors from the ChemDIV Database. B) Docking models were developed based on the actived pocket of NSUN2 crystal structure and the 2091804 compounds. C) Docking pose of the top 5 inhibitors within the catalytic center of NSUN2 protein (left). 2D structure of these 5 small‐molecule inhibitors (right). D) CCK8 analysis of the CRC cells after treatment with small molecule inhibitor Nsun2‐i4. E) MST analysis of the interactions between Nsun2‐i4 inhibitor and NSUN2 protein. F) Immunoblot for the indicated proteins in SW480 and HT29 cell lines following exposure to 15 µM Nsun2‐i4 for 12 h. G) Schematic of the administration of small molecule inhibitors (Nsun2‐i4) in C57BL/6N mice (left). HE staining of liver, spleen, kidney and colon in mouse model treated with Nsun2‐i4 (right). H) Schematic of small molecule inhibitors (Nsun2‐i4) in AOM/DSS‐induced orthotopic mouse model of CRC. I) Typical images of colorectal tumors from control and Nsun2‐i4‐treatment groups of mice in AOM/DSS‐induced orthotopic CRC model (left). Comparison of the total of colorectal tumor numbers in mice from control and Nsun2‐i4 groups (middle). Comparison of the indicated size of colorectal tumor numbers in mice from control and Nsun2‐i4 groups (right). J) Schematic diagram showing the combined therapy effect of small molecule inhibitors (Nsun2‐i4) and PD‐1 in syngeneic C57BL/6N mice. K) Images of the dissected subcutaneous tumors from the tumor‐bearing mice at the end of syngeneic C57BL/6N model (n= 5 mice per group). L) Time‐course evaluation of subcutaneous tumor volume measured every 4 days in C57BL/6N mice. M) The final weight of the subcutaneous tumors was shown in the scatter plot for indicated groups.

Given that the apparent role in NSUN2‐driven tumorigenesis, we next performed structure‐based virtual screening of 2,091,084 compounds from the ChemDiv library to identify potential NSUN2 inhibitors for the improvement of clinical usability (**Figure** **6**A). From ChemDiv, we requested the top 62 candidate compounds that exhibited the highest scores in their docking to the catalytic pocket of NSUN2 (Figure 6A,B). The top five compounds were selected and further validated in CRC cells using cell counting kit‐8 (CCK‐8) cell proliferation/viability assay (Figure 6B–D). According to these results, Nsun2‐i4 demonstrated inhibitory effects in two CRC cell lines, with the lowest IC50 values measured at 56.04 µM in SW480 cell and 45.79 µM in HT29 cell (Figure 6D). Therefore, Nsun2‐i4 was selected for further validation. The molecular docking analysis, based on the crystal structure of NSUN2, indicated that Nsun2‐i4 interacted with distinct NSUN2 residues, including Tyr83, Hie86, Asn157, Gln605, and Glu694 (Figure S6A, B, Supporting Information). Supporting this model, MST assay demonstrated that Nsun2‐i4 bound to the NSUN2 protein with a KD of 2.65 ± 1.73 µM (Figure 6E). Moreover, treating CRC cells with 50 µM Nsun2‐i4 for 12 h remarkably decreased the expression levels of ENO1 and GRB2 proteins (Figure 6F). The adminstration of Nsun2‐i4 to control CRC cells resulted in a significant reduction in *ENO1* mRNA expression, whereas no significant change was observed in *ENO1* mRNA levels when Nsun2‐i4 was added to *NSUN2*‐knockout CRC cells (Figure S6C, Supporting Information). The half‐life of *ENO1* RNA was markedly decreased in CRC cells followed by the treatment of Nsun2‐i4 (Figure S6D, Supporting Information). Moreover, the decreased m^5^C abundance was confirmed by dot‐blot assay of RNA samples from Nsun2‐i4‐treated CRC cells (Figure S6G, Supporting Information). To demonstrate the safety and applicability of this small‐molecule NSUN2 inhibitor, in vivo experiments were conducted in mice to evaluate its systemic toxicity, vital organ toxicity, and therapeutic efficacy. Chronic treatment of C57BL/6N mice with Nsun2‐i4 did not result in any significant changes in body weight, or the histopathological analyses of major organs, including the kidney, liver, spleen, and colon, when compared with those of the control group, indicating that Nsun2‐i4 treatment does not cause obvious toxicity in vivo (Figure 6G; Figure S6E,F, Supporting Information). We then established an AOM/DSS‐induced model, in which mice were administered Nsun2‐i4 (50 mg/kg intraperitoneally), to further assess the role of this small‐molecule inhibitor in CRC tumorigenesis (Figure 6H). These results indicated that Nsun2‐i4 effectively inhibited tumor growth rates and reduced the total tumor burden in AOM/DSS‐induced CRC (Figure 6I). Recent studies have reported that NSUN2 activation maintains global m^5^C RNA methylation of several oncogenes, and promotes tumorigenesis and immunotherapy resistance via cGAS‐STING pathway.^[^ ^41^ ^]^ In accordance with this, significant cGAMP‐induced activation of p‐STING and p‐TBK1 were observed in CRC cell when treated with Nsun2‐i4 (Figure S6H, Supporting Information). Furthermore, immunohistochemistry analysis in immunocompetent mice confirmed notable activation of the cGAS‐STING pathway following Nsun2‐i4 treatment (Figure S6I, Supporting Information). Hence, we evaluated the potential synergy between Nsun2‐i4 and PD‐1 in mouse model (Figure 6J). The results showed that treatment with either compound alone effectively reduced subcutaneous tumor growth; however, co‐administration of PD‐1 with Nsun2‐i4 in combination demonstrated a significantly enhanced inhibitory effect on tumor growth compared with individual treatments (Figure 6K–M). Overall, these findings showcase  NSUN2 as a promising target for cancer immunotherapy in combination with immune checkpoint blockade.

### 2.8 NSUN2 and ENO1 are Correlated with Glucose Metabolism in CRC Patients

**Figure 7:**
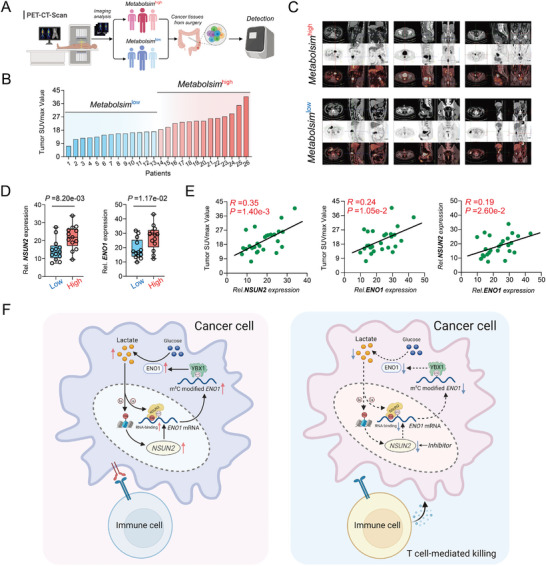
NSUN2 and ENO1 are correlated with glucose metabolism in CRC patients. A) Schematic diagram of integration of clinical CRC patient tissues with PET‐CT results and the analysis of glucose metabolism. B) The ranking chart of CRC patients based on glucose metabolism as per PET‐CT SUV_max_ values. C) Representative PET‐CT images of CRC patients categorized into glucose metabolism^high^ and metabolism^low^ groups. D) The relative RNA expression levels of *NSUN2* (left) and *ENO1* (right) in metabolism^high^ and metabolism^low^ groups. E) The correlation analysis of the relative RNA expression level of *NSUN2* and tumor SUV_max_ (left). The correlation analysis of the relative RNA expression level of *ENO1* and tumor SUV_max_ (middle). The correlation analysis of the relative RNA expression levels of *NSUN2* and that of *ENO1* in CRC patients (right). F) Schematic summarization of key findings presented in this study.

Studies have shown that 18 fluorodeoxyglucose positron emission‐computed tomography (PET‐CT) can be utilized to visualize tumor biology and assess in vivo tumor glucose metabolism levels, of which SUV_max_ is the most commonly employed quantitative indicator of glucose uptake.^[^ ^42^ ^]^ Based on the value of SUV_max_ derived from PET‐CT, 26 individuals diagnosed with CRC were divided into  metabolism^high^ and metabolism^low^ groups (**Figure** **7**A–C; Table S6, Supporting Information). In accordance with the aforementioned findings, the analysis of clinical samples validated the higher expression of both NSUN2 and ENO1 in the metabolism^high^ group than that of metabolism^low^ group (Figure 7D). Moreover, our findings indicated statistically significant positive correlation between the RNA expressions of either *NSUN2* or *ENO1* and SUV_max_ values (Figure 7E). Among these patients, the RNA expression of *ENO1* exhibited a notable and statistically significant positive correlation with that of *NSUN2* (Figure 7E). Then, we explored the potential interaction between the glycolysis level and the expression levels of NSUN2 and ENO1 in influencing patient survival outcomes. Within the TCGA‐CRC cohorts, we stratified patients into distinct subgroups based on their NSUN2 and ENO1 expression levels, along with the glycolysis level. Survival analysis indicated that the CRC patients in the Glycolysis^low^/NSUN2^low^/ENO1^low^ signature subgroup exhibited superior overall survival (OS) compared to those with Glycolysis^high^/ NSUN2^high^/ENO1^high^ signature subgroup (Figure S7A, Supporting Information). Parallel analyses were performed for disease‐specific survival (DSS) and progression‐free survival (PFS), yielding results that align closely with those observed for OS (Figure S7B,C, Supporting Information). Therefore, we substantiated in clinical patients that NSUN2‐mediated reprogramming of glucose metabolism through ENO1 functions as a contributory risk factor for CRC.

## 3 Discussion

Abnormal m^5^C modification has been implicated in several diseases, encompassing cancer, inflammation, neurodevelopmental disorders, intellectual disabilities, infertility, and mitochondrial dysfunction. In our previous study, we found that RNA m^5^C modification may play an important role in CRC tumorigenesis.^[^ ^34^ ^]^ Specifically, the m^5^C score system confirmed that CRC tissues exhibited significantly elevated m^5^C scores compared with adjacent normal tissues. Moreover, increased m^5^C scores were observed in BLCA, LIHC, ESCA, and STAD, in which the mechanisms of m^5^C modification in carcinogenesis have been elucidated, firmly demonstrating the validity of this scoring system.^[^ ^10^, ^13^, ^17^, ^18^, ^19^ ^]^ However, the precise relationship between the m^5^C regulatory genes and cancer development remains unclear. In the present study, the 18 m^5^C regulatory genes were included in the rank analysis of the 32‐pair independent clinical CRC cohort from the hospital and found that NSUN2 exhibited the highest fold change with a significant *P* value. NSUN2 is a well‐characterized RNA methyltransferase, responsible for m^5^C of tRNA and mRNAs.^[^ ^10^, ^13^, ^17^, ^18^, ^19^, ^35^ ^]^ NSUN2 promotes the progression of multiple cancers via m^5^C modification of *LIN28B*, *FABP5*, *p57^Kip2^*, *PFAS*, *TIAM2*, and *HDGF* mRNA in esophageal squamous cell carcinoma, osteosarcoma, gastric cancer, retinoblastoma, pancreatic cancer and bladder cancer, respectively.^[^ ^10^, ^18^, ^43^, ^44^, ^45^, ^46^ ^]^ Correlation analysis further revealed that high levels of *NSUN2* were significantly associated with clinical stage, lymph node metastasis, and distant metastasis. To verify these findings, colony formation, transwell invasion, and tumor sphere formation assays demonstrated that NSUN2 knockout significantly suppressed the proliferation, invasion, and cancer stemness of CRC cells. In accordance with in vitro results, subcutaneous tumor, tail vein injection lung metastasis, and intra‐spleen injection liver metastasis models were established, and the results indicated that NSUN2 knockout significantly inhibited subcutaneous tumor growth and distant metastasis. The genetically engineered mouse orthotopic CRC model also indicated that the NSUN2 heterozygotes exhibited significantly less tumor burden in AOM/DSS‐induced CRC than the control. Taken together, these in vitro and in vivo results demonstrated the crucial role of *NSUN2* in CRC development and metastasis.

To explore the underlying mechanisms by which NSUN2 regulates the progression and metastasis of CRC, transcriptome sequencing and bisulfite sequencing were conducted. We found that NSUN2 knockout significantly reduced glucose metabolic processes in CRC, and identified ENO1 as the downstream target of NSUN2 in an m^5^C‐independent manner. ENO1,  the core catalytic enzyme of glycolysis, has been implicated in tumor initiation and progression of numerous cancers through canonical metabolic pathways or non‐metabolic atypical pathways, including CRC and LIHC.^[^ ^47^, ^48^, ^49^, ^50^, ^51^, ^52^ ^]^ Typically, m^5^C‐modified RNA is recognized by reader proteins, allowing it to exert its functions such as enhancing RNA stability in an m^5^C‐dependent manner.^[^ ^10^, ^18^ ^]^ Subsequent mass spectrometry analysis was conducted, identifying YBX1 as a mediator of m^5^C‐*ENO1*. YBX1, a well‐known m^5^C‐binding reader protein, functions alongside NSUN2 to enhance the mRNA stability of *HDGF* in BLCA and the mRNA translation of *QSOX1* in non‐small‐cell lung cancer.^[^ ^10^, ^11^ ^]^ Then, the analysis of TCGA data further validated the significantly positive correlation between the mRNA expression of *ENO1* and those of *NSUN2* and *YBX1* in CRC. In summary, these results indicated that NSUN2 and YBX1 served as the m^5^C “writer” and “reader” of *ENO1*, thereby regulating metabolic reprogramming and allowing the enhancement of glucose metabolism.

Cancer cells exhibit a predilection for metabolizing glucose via glycolysis, even when ample oxygen is available, a phenomenon commonly referred to aerobic glycolysis or the Warburg effect.^[^ ^53^ ^]^ Lactic acid, formerly regarded as the byproduct of glycolysis, can modify the lysine residues of histones, resulting in a novel epigenetic modification known as lactylation.^[^ ^28^, ^29^, ^30^, ^31^, ^32^, ^33^ ^]^ Histone H3 lysine lactylation, induced by lactic acid in macrophages, not only alters the macrophage polarization state by increasing the expression of *Arg‐1 *and other M2‐like macrophage genes, but also exhibits positive correlation with oncogenic production by reparative M2 macrophages.^[^ ^54^ ^]^ In addition, the lactylated METTL3 potently induces the immunosuppressive effects of tumor‐infiltrating myeloid cells via JAK1‐STAT3 signaling pathway in an m^6^A‐dependent manner.^[^ ^29^ ^]^ In the current study, we found that lactic acid promoted histone H3K18la, thereby activating *NSUN2* gene transcription. In addition, single‐point mutation and immunoprecipitation assays demonstrated that K356, rather than K556 or K511, of the NSUN2 protein directly binds to lactic acid, which is essential for capturing target m^5^C modified RNAs. Herein, we uncovered an interesting positive feedback loop in which NSUN2 promoted and stabilized *ENO1* mRNA, and then enhanced glycolysis and increased lactic acid production resulting from the upregulated ENO1, which in turn stimulated NSUN2 transcription. Hence, targeting NSUN2 may disrupt this positive feedback loop. Recent advances have shown that lactic acid acts as a metabolic checkpoint for Treg cells and controls immune responses in the TME via the upregulation of PD‐1 expression. In addition, studies have established that NSUN2 activation maintains global m^5^C RNA methylation, and stabilizes *TREX2* to restrict cytosolic dsDNA accumulation and cGAS/STING activation, ultimately promoting tumorigenesis and fostering resistance to anti‐PD‐L1 immunotherapy.^[^ ^41^ ^]^ To enhance the potential clinical applicability of this research, molecular docking‐based virtual screening was used to screen out the small‐molecule inhibitor of NSUN2 from the ChemDIV Database, and Nsun2i‐4 was identified as an effective inhibitor without obvious toxicity in vivo. Hence, the CRC syngeneic mouse model was employed to further assess the efficacy of NSUN2 inhibitor in combination with PD‐1 blockades, and the encouraging results supported the potential ability of NSUN2 for cancer immunotherapy in CRC. Ultimately, the reliability of the regulatory axis was validated through the analysis of an independent clinical CRC cohort using PET‐CT and large‐scale genomic sequencing data from TCGA database. Indeedly, our independent clinical cohort results and TCGA data analysis further corroborated the higher expression levels of NSUN2 and ENO1 in the high‐glucose metabolism group, and the patients with Glycolysis ^low^/NSUN2^low^/ENO1^low^ signature showed substantial survival benefit compared to that of patients with glycolysis Glycolysis^high^/NSUN2^high^/ENO1^high^ signature in the TCGA‐CRC cohort, respectively.

In conclusion, our substantial body of work focusing on the NSUN2/YBX1/m^5^C‐ENO1 signaling axis may offer valuable insights pertaining to the pathogenesis and identification of epigenetic–immune targets in CRC.

## 4 Experimental Section

### Bioinformatics Analysis

Transcriptomic data and clinical information for CRC and noncancerous samples were obtained from TCGA data portal (https://portal.gdc.cancer.gov). The ClusterProfiler R package was employed for GO analysis and KEGG analysis, while the Survival package and Corrplot package in R were utilized for performing survival analysis and pearson correlation analysis, respectively, as detailed in previous descriptions. The m^5^C scroes were established by the m^5^C regulatory genes according to the GSCA platform as previously described.^[^ ^55^ ^]^

### Cell Culture

CRC cell lines and human embryonic kidney HEK 293T cells were obtained from ATCC. All cell lines were maintained using standard cell culture procedures as previously described.^[^ ^56^, ^57^ ^]^

### Transwell and Colony Formation Assay

The transwell and colony formation assays were conducted following previously established procedures.^[^ ^56^ ^]^ In short, the CRC cells treated with various transfections were seeded in the upper chamber pre‐coated with matrigel for 48 h. Following this incubation, the cells that invaded the undersurface of the membrane were fixed and quantified under the optical microscope. In the colony formation assay, CRC cells were seeded and cultured for 14 days, after which the total number of cell colonies, each containing more than 50 cells, was tallied and photographed.

### Sphere Formation Assay

As previously described, the sphere formation assay was conducted with slight adjustments. The CRC cells was planted into ultra‐low cluster plate in serum‐free DMEM/F12 (1:1) medium (Gibco) containing growth factors (EGF 20 ng/mL and bFGF 10 ng/mL; PeproTech), 2% B‐27 supplement (Gibco), 2 µg/mL of 0.2% heparin (Solarbio). After 14 days, the observation of sphere formation was conducted using an inverted microscope, and photographs were taken for subsequent analysis.

### Specimen Collection and Ethics Approval

In total, 126 patients, diagnosed with CRC and undergoing surgical resection at Zhongnan Hospital of Wuhan University between September 2017 and December 2022, were included in this study. All participants provided written informed consent, and the study design was approved by the Ethics Committee of Zhongnan Hospital of Wuhan University. Detailed information pertaining to these CRC patients is available in Table S1 (Supporting Information).

### RNA Extraction and RT‐qPCR

Total RNA extraction, cDNA reverse transcription, and RT‐qPCR analysis were conducted according to established protocols, as previously detailed.^[^ ^56^, ^57^ ^]^ Sequences of the specific forward and reverse primers used in this study are summarized in Table S2 (Supporting Information).

### IP and Western Blot Assays

IP and western blotting assays were conducted following previously established protocols.^[^ ^56^, ^57^ ^]^ The primary and secondary antibodies utilized in this study are detailed in Table S7 (Supporting Information).

### Animal Model

Animal studies received approval from the Institutional Animal Care and Use Committee, and all mice were kept at the Wuhan Institute of Virology, Chinese Academy of Sciences (Wuhan, China), after undergoing a rigorous and equitable randomization process. All animal tumor models were established as previously described, with some minor modifications. In brief, *Ctrl* or *NSUN2* ^KO^ CRC cells were implanted subcutaneously on the back of each nude mouse or injected into the caudal vein for the subcutaneous tumor model and lung metastasis model, respectively. To establish the CRC liver metastasis mouse model, *Ctrl* or *NSUN2* ^KO^ CRC cells (2 × 10^6^) were injected into the distal spleen of each nude mouse after anesthetization with an intraperitoneal injection of 2.5% Avertin. Fifteen minutes later, the blood vessels of the distal spleen were ligated, the distal spleen was excised, and the abdominal wall was sutured. After six weeks, the mice were sacrificed and dissected, after which liver weight and liver metastasis nodules were measured. The AOM/DSS murine model of CRC tumorigenesis was developed as previously described. The *Nsun2* ^+/−^ C57BL/6N mice were purchased from Saiye Biotechnology Co., Ltd. Briefly, 8‐week‐old WT C57BL/6N mice or *Nsun2^−/−^* C57BL/6N mice were subjected to intraperitoneal injections of 10 mg/kg AOM (Sigma‐Aldrich). A week later, the mice received drinking water containing 2.5% DSS (MP Biomedicals, Santa Ana, CA, USA) every day for seven days, followed by undergoing a two‐week recovery period with regular drinking water. The same induction and maintenance cycles were repeated twice, and on day 105, the mice were euthanized to assess tumor burdens.

### RNA‐BiS‐Seq

The RNA‐BiS libraries were constructed by E‐GENE Co. Ltd as follows. In brief, the extracted total RNA underwent initial DNase I treatment at 37°C for 30 min to eliminate residual DNA. Following purification, mRNA enrichment was performed using the Dynabeads mRNA Purification Kit (Thermo Fisher Scientific, USA), following the manufacturer's instructions. The isolated mRNA samples underwent RNA bisulfite conversion using the EZ RNA Methylation Kit (Zymo Research, USA), involving an initial step at 70 °C for 5 min, followed by incubation at 54 °C for 45 min. Subsequently, purification was carried out using the supplied Zymo‐Spin IC Column, following the manufacturer's instructions. The reverse transcription of bisulfite‐converted RNA utilized ACT random hexamers and Superscript II Reverse Transcriptase (Invitrogen, USA). The resulting cDNA was subsequently employed for the construction of the final library. After analysis with the Agilent 2100 Bioanalyzer (Agilent Technologies, USA) and quantification via real‐time PCR, the RNA‐BiS libraries were ultimately sequenced using the Illumina Novaseq 6000 platform (Illumina, USA).

### RNA‐Seq

Briefly, total RNA from each sample was extracted using Invitrogen TRIzol Reagent, followed by 30‐minutes treatment with RNase‐free DNase I in accordance with the manufacturer's protocols. Poly (A)‐containing mRNA was isolated by employing Oligo Beads with about 1 µg of total RNA. The captured mRNA was initially fragmented into 100–200 nt using divalent cations at an elevated temperature. The fragmented mRNA underwent reverse transcription with SuperScript II and was then converted into double‐stranded cDNA using RNaseH and DNA Pol I through random priming. Following purification, the double‐stranded cDNA underwent processes such as blunt‐ending, dA addition to the 3’‐end, and adapter ligation. Then, PCR was conducted to enrich the adapter‐ligated cDNA. The libraries were then analyzed using the Agilent Bioanalyzer 2100, quantified via qPCR, and finally sequenced using the Illumina sequencing platform.

### MST

The Kd of binding of *ENO1* [m^5^C] or *ENO1* [C] to the YBX1 protein and Nsun2‐i4 or Nsun2‐i5 to the NSUN2 protein was assessed using a Monolith NT.115 instrument (NanoTemper Technologies, Germany). Prior to testing, all the protein samples were solubilized in RIPA buffer. YBX1‐GFP and NSUN2‐GFP were added to each dilution and incubated at room temperature for 5 min. After loading the samples into silica capillaries, the measurements were carried out at 25 °C with 60% LED power and medium MST power. The data were analyzed using Nano‐Temper Analysis software, version 2.3.

### RNA Stability Assay

RNA stability measurements were conducted as previously outlined.^[^ ^57^ ^]^ The CRC cells were transfected with the indicated vectors and subsequently treated with actinomycin D (5 µg/mL) for the specified duration. Following treatment, the cells were harvested, and total RNA was extracted and subjected to RT‐qPCR. The mRNA half‐life was determined using linear regression analysis.

### PAR‐CLIP

PAR‐CLIP was conducted in accordance with a previously published method, with some modifications.^[^ ^10^ ^]^ SW480 and HT29 cells, following the specified treatments, were cultured in medium supplemented with 100 µ 4‐thiouridine (Sigma, USA) for 14 h and subsequently crosslinked with UV light. Using the PAR‐CLIP‐biotin chemiluminescent nucleic acid detection protocol, we biotin‐labeled the protein‐RNA‐bead complex utilizing an RNA 3' end biotinylation kit (Thermo Fisher Scientific, USA) and then visualized it using the chemiluminescent nucleic acid detection module.

### Vectors, RNA Interference

Human shRNAs (Table S5, Supporting Information) were expressed in the PLKO.1 vector. Mutations in the NSUN2 sequences were generated by overlap extension PCR. The siRNA sequences were designed and provided in Table S5 (Supporting Information). Lipofectamine 2000 (Invitrogen, USA) was employed to transfect the siRNA or plasmid following the manufacturer's instructions.

### Extraction of Cytoplasmic and Nuclear Lysates

Cytoplasmic and nuclear lysates were extracted to investigate the localization of NSUN2, following previously described procedures. In summary, cells were cold‐lysated on ice using cell fractionation buffer for 10 min, followed by centrifugation at 500 g for 5 min to separate the cytoplasmic fraction. The resulting precipitate was lysed in cell disruption buffer to extract the nuclear proteins.

### RNA‐Affinity Chromatography, RNA Pull‐Down, and Mass Spectrometry Analysis

RNA‐affinity chromatography was conducted in accordance with previously described methods.^[^ ^18^ ^]^ Biotin‐labeled RNA fragments, including 50‐bp ENO1 RNA sequences with m^5^C modification (ENO1 [m^5^C]) or without m^5^C modification (ENO1 [C]) at the m^5^C site, were synthesized (as detailed in Table S5, Supporting Information) and then incubated with protein extracts from CRC cells. In brief, biotinylated RNAs, up to 50 pmol, were blended with 2 mg of protein lysates and 50 µL of streptavidin beads. Following the RNA pull‐down assay, the proteins were isolated and analyzed using mass spectrometry or western blotting.

### ChIP‐qPCR

According to the manufacturer's instructions, ChIP analysis was performed with an anti‐H3K18la antibody using the ChIP Assay Kit. Fold enrichment was calculated through RT‐qPCR analysis and expressed as a percentage of the input chromatin (Input %). The sequences of specific primers are detailed in Table S2 (Supporting Information).

### CCK‐8 Assay

The CCK‐8 assay was conducted following previously described procedures.^[^ ^56^, ^57^ ^]^ In brief, CRC cells were plated in 96‐well plates, and various inhibitors at different concentrations were introduced to the cells after 24 h. At the 96‐hour time point, 10 µL per well of CCK‐8 solution was added, followed by incubation for 2h. Finally, the optical density was measured using a microplate reader (Bio‐Rad Laboratories, Hercules, CA, USA).

### Lactate Production and Glucose Consumption Assessments

Lactate production and glucose consumption were evaluated using a Lactate Assay kit (Sigma‐Aldrich, USA) and a Glucose Uptake Colorimetric Assay Kit, respectively, following the manufacturer's protocols. For the glucose uptake assay, CRC cells were initially placed in the 96‐well culture plate and incubated in Glucose Uptake Buffer (AAT Bioquest, USA) for 1 h, after which they were exposed to 10 µL of 2‐DG (AAT Bioquest, USA) per well for 40 minutes. Finally, 50 µL of the Uptake Assay Mixture (AAT Bioquest, USA) was added, and the samples were assessed using a microplate reader (Tecan Group Ltd., Switzerland) at excitation/emission wavelengths of 570/610 nm. During the L‐lactate assay, each sample was incubated with 50 µL of the L‐Lactate working solution (AAT Bioquest, USA) for 2 h. The samples were measured at 575 nm/605 nm using a microplate reader, and the lactate concentrations were measured based on the lactate standard curve.

### Measurement of ECAR

Real‐time measurements of ECAR in CRC cells were conducted using an XFe96 Extracellular Flux Analyzer (Agilent Technologies, USA). In brief, the CRC cells were seeded in the medium containing 2 mmol/L glutamine and 10 mmol/L glucose at a density of 5 × 10^4^ cells per well. After temperature and pH equilibration, measurements were taken using the analyzer following the manufacturer's protocol.

### Docking and Molecular Modeling

The computer‐based virtual docking utilized the crystal structure based on NSUN2 (PDB: Q08J23), wherein 2.09 million small molecules from the ChemDiv database were screened. Schrodinger's Ligprep program was employed to conduct hydrogenation and energy optimizations for the molecules in the database. The preprocessing of protein structures was carried out using Schrödinger's Protein Preparation program. The database underwent a cascade docking procedure using Schrödinger's Glide module, which encompassed HTVS, SP, and XP docking steps. Ultimately, this selection process led to the identification of the top five candidate small molecules (Table S8, Supporting Information) for further evaluation in cellular experiments. The m^5^C‐*ENO1* RNA complex structure was modelled by molecular docking using HDOCK. Structures were visualized using PyMol (version 1.8.6.0). The RING software was utilized for the analysis of the residue interaction network.

### Luciferase Reporter Gene Assays

Luciferase activity was measured as previously described.^[^ ^56^, ^57^ ^]^ ENO1‐WT or ENO1‐MUT (cytosine‐to‐guanine substitution at the m^5^C site) was cloned downstream of the pmirGLO vector. Then, these constructed vectors were co‐transfected with the pRL‐SV40 Renilla vector into CRC cells with or without YBX1 overexpression in 24‐well plates. Relative luciferase activity was evaluated 48 h after transfection using the Dual‐Luciferase Reporter Assay System(Promega). The results were normalized to Renilla luciferase activity.

### m^5^C Dot Blot Assay

m^5^C dot blot assay was conducted as previously described.^[^ ^41^ ^]^ CRC cells were cultured and treated with Nsun2‐i4 for 12 hours, followed by collection and extraction of total RNA. The indicated amounts of RNA samples were then spotted onto a nylon membrane and subjected to UV crosslinking for 25 min. Subsequently, the membrane was blocked in 5% milk in TBST buffer for 1 h and incubated with the m^5^C antibody overnight at 4 °C. After washing with TBST buffer, the membrane was incubated with a secondary antibody conjugated with horseradish peroxidase at room temperature for 1 h, followed by further washing with TBST buffer. Chemiluminescent substrate was used for signal development, while a loading control was established by staining the membrane with 0.1% Methylene Blue (MB) and subsequent washing with ddH_2_O.

### Statistical Analysis

Statistical analyses were conducted in SPSS software (version 22.0; SPSS, Inc., USA), and the results were visualized using GraphPad Prism software (version 7.0) and R (version 3.6.2). Continuous variables, presented as the mean ± standard deviation (SD), were subjected to unpaired Student's t‐tests for comparing the means of two groups with equal variances. Survival analysis was conducted using the Kaplan–Meier method, and comparisons were made using a log‐rank test. Pearson correlation analysis provided the correlation coefficient (r) and *P* values < 0.05 indicated statistical significance (*P < 0.05, **P < 0.01, ***P < 0.001).

## Conflict of Interest

The authors declare no conflict of interest.

## Author Contributions

B.C., Y.D., Y.H., and L.F. contributed equally to this work. B.C., J.Z., Y.H., X.R., X.X., and C.J. designed the study. B.C., J.Z., and Y.D. carried out the most experiments. H.H., X.Z., and S.Y., contributed patient samples and clinical information. B.C., J.Z., and X.Z. performed bioinformatic analysis. H.H., X.Z., and S.Y. conducted the statistical analysis. B.C., J.Z., and X.R. wrote the manuscript. Q.C., X.R., J.Z., and C.J. revised the paper. All authors read and approved the final manuscript.

## Supporting information

Supporting Information

## Data Availability

The data that support the findings of this study are available from the corresponding author upon reasonable request.
