# Supplementary material for: Metabolic Recoding of NSUN2‐Mediated m5C Modification Promotes the Progression of Colorectal Cancer via the NSUN2/YBX1/m5C‐ENO1 Positive Feedback Loop
Source: Adv Sci (Weinh). 2024 May 20;11(28):2309840. doi: 10.1002/advs.202309840 (PMC11267267; doi:10.1002/advs.202309840)
Supplement: Supplementary file 1 — Supporting Information [file ADVS-11-2309840-s001.docx]

Figure S1

**
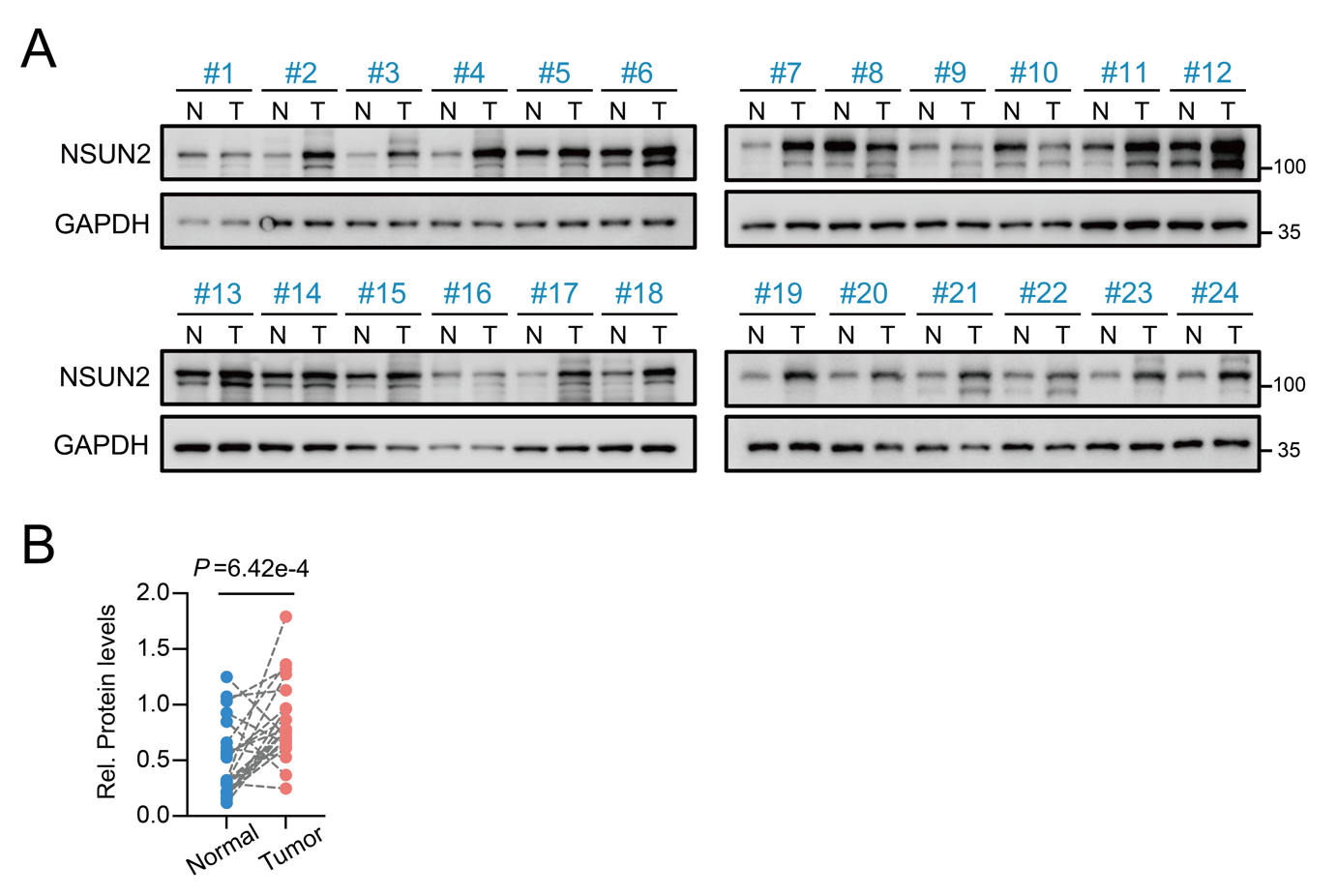
**

Figure S1. NSUN2 was observed to be upregulated in CRC tissues compared to adjacent non-cancerous tissues. (A) WB analysis of protein levels of NSUN2 in 24 pairs of clinical CRC specimens. (B) Statistical analysis of NSUN2 protein expression in 24 pairs of clinical CRC and adjacent tissues.

Figure S2

**
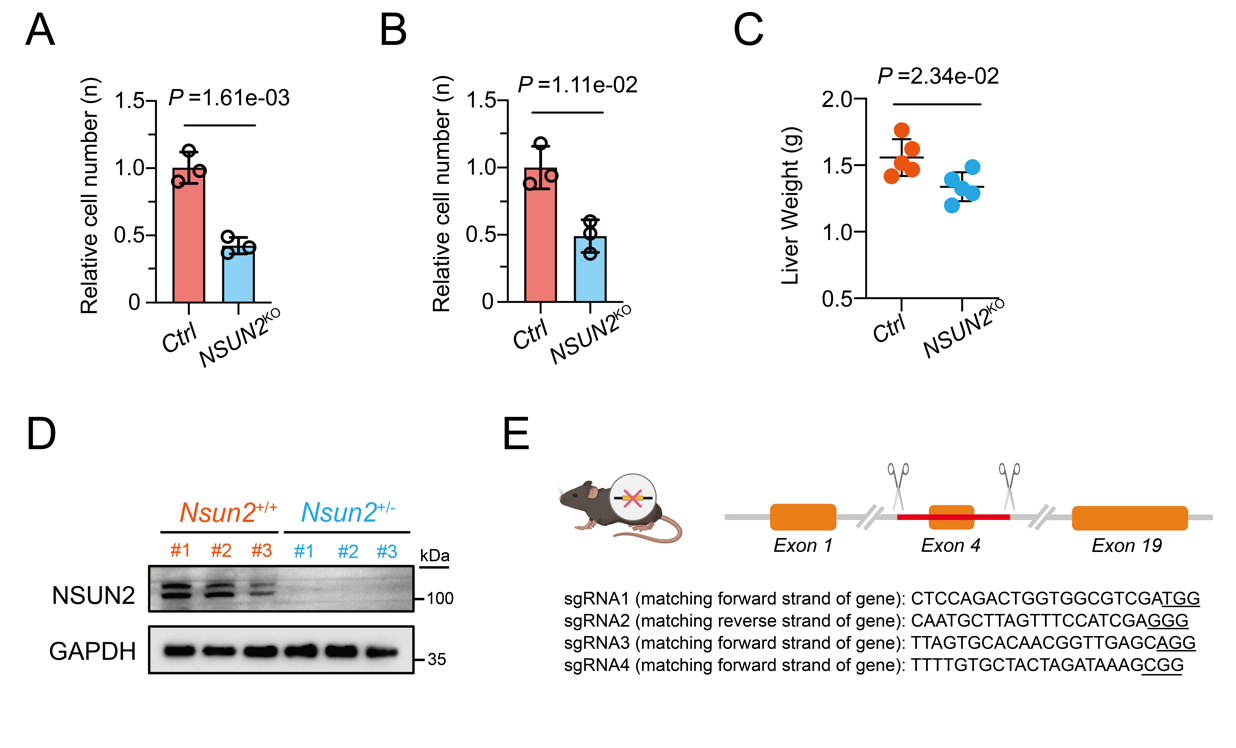
**

Figure S2. NSUN2-deficency impedes the tumorigenesis and metastasis of CRC *in vitro* and *in vivo*. (A) Statistical analysis of the transwell assay in SW480 cell for three independent experiments. (B) Statistical analysis of the colony formation assay in SW480 cell for three independent experiments. (C) Statistical analysis of the liver weight in both control and *NSUN2* knockout groups of the intra-splenic liver metastasis mouse model of CRC. (D) Schematic diagram of the construction of *Nsun2* knockout mice. (E) Western blot analysis of the protein expression of NSUN2 in *Nsun2*^+/+^ and *Nsun2*^-/-^ mice.

Figure S3

**
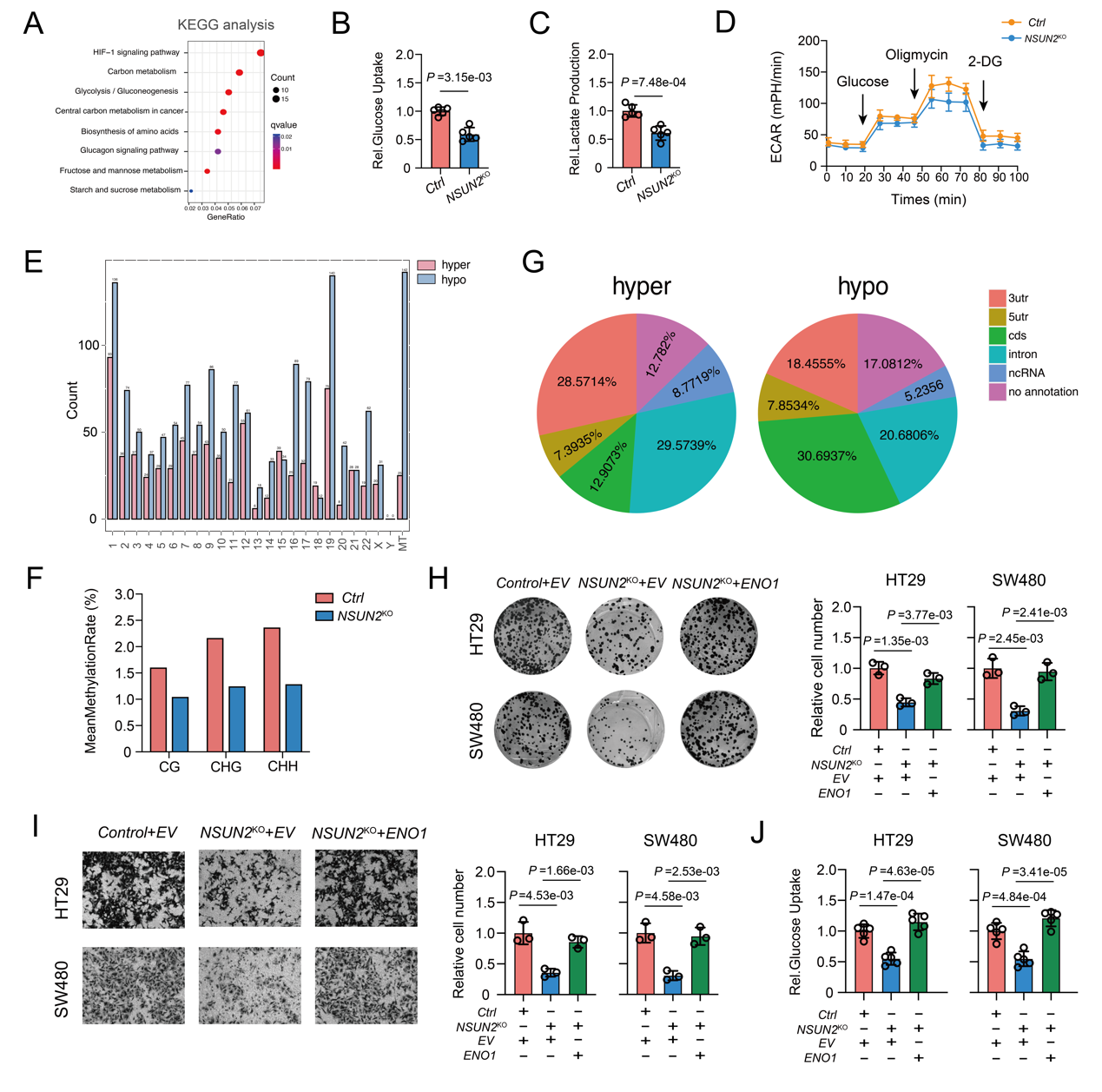
**

Figure S3. NSUN2-mediated m^5^C modification leads to the reprogramming of glucose metabolism by targeting ENO1 in CRC. (A) KEGG analysis of the genes with differential expression between the control and *NSUN2* knockout CRC cells. (B) Comparison of the relative glucose uptake between control and *NSUN2* knockout HT29 cells. (C) Comparison of the relative lactate production between control and *NSUN2* knockout HT29 cells. (D) Analysis of ECAR in control and *NSUN2* knockout HT29 cells. (E) The distribution of chromosomes for genes with increased (hyper) and decreased (hypo) methylation when *NSUN2* was knocked out. (F) The alteration in RNA m^5^C site levels identified in each sequence context (CG, CHG, and CHH, where H=A, C, or U) in both control and *NSUN2* knockout CRC cells. (G) The distribution of gene regions for genes exhibiting increased (hyper) and decreased (hypo) methylation when *NSUN2* was knocked out. (H) Representative images and quantification of the rescue colony formation results in HT29 and SW480 cells. (I) Representative rescue transwell assay of HT29 and SW480 cells (left), and the accompanying bar graphs display the relative colony numbers (right). (J) The rescue experiment of glucose uptake subsequent to the overexpression of ENO1 in *NSUN2*-knockout CRC cells.

Figure S4

**
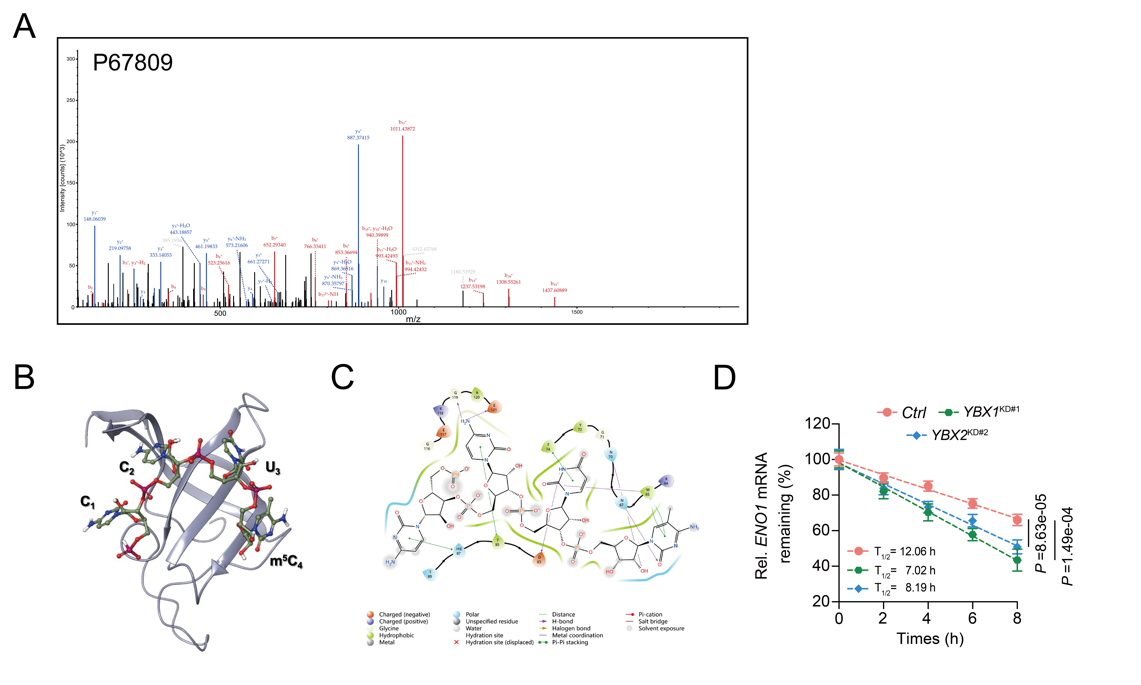
**

Figure S4. YBX1 acts as an m^5^C “reader”, identifying and stabilizing *ENO1* mRNA. (A) The mass spectrogram of YBX1 (P67809) identified by *ENO1* m^5^C RNA oligo. (B-C) An overall view of the YBX1 CSD domain in complex with the *ENO1* m^5^C RNA oligo. The YBX1 CSD domain is displayed as a purple ribbon, and the *ENO1* m^5^C RNA oligo is shown as a stick model. (D) Effects of YBX1 knockdown on mRNA half-life of *ENO1* by RNA stability assays

Figure S5

**
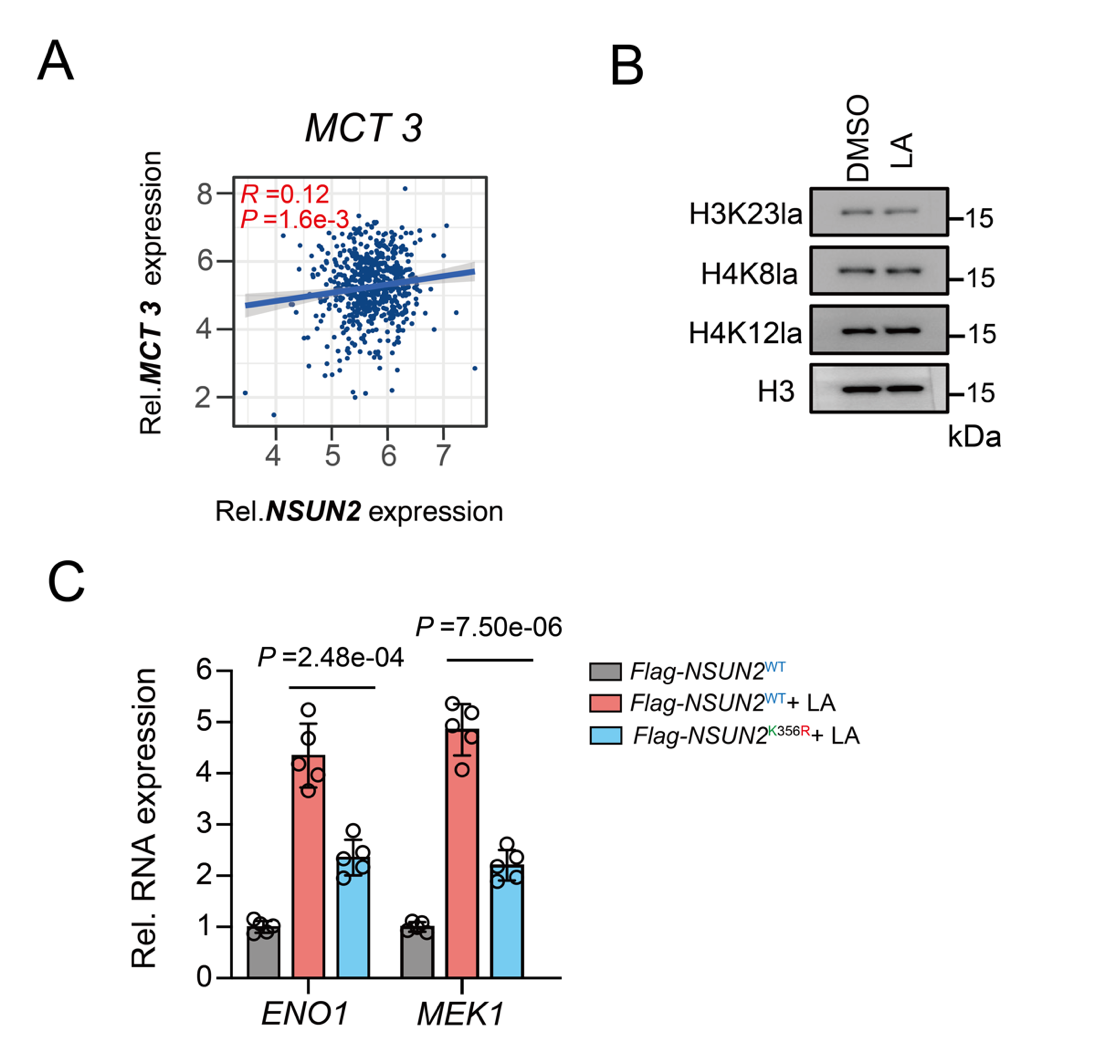
**

Figure S5. Tumor-derived lactic-acid activates the transcription of NSUN2 through histone H3K18la and directly induces the lactylation of NSUN2 at the K356. (A) The correlation analysis of the relative mRNA expression levels between *NSUN2* and *MCT3* in TCGA-CRC database. (B) WB analysis of the indicated protein levels from the whole-cell lysate of CRC cells with addition of 25 mM L-lactic acid. (C) RT-qPCR analysis of the relative RNA expression levels in SW480 cellswith different treatment.

Figure S6

**
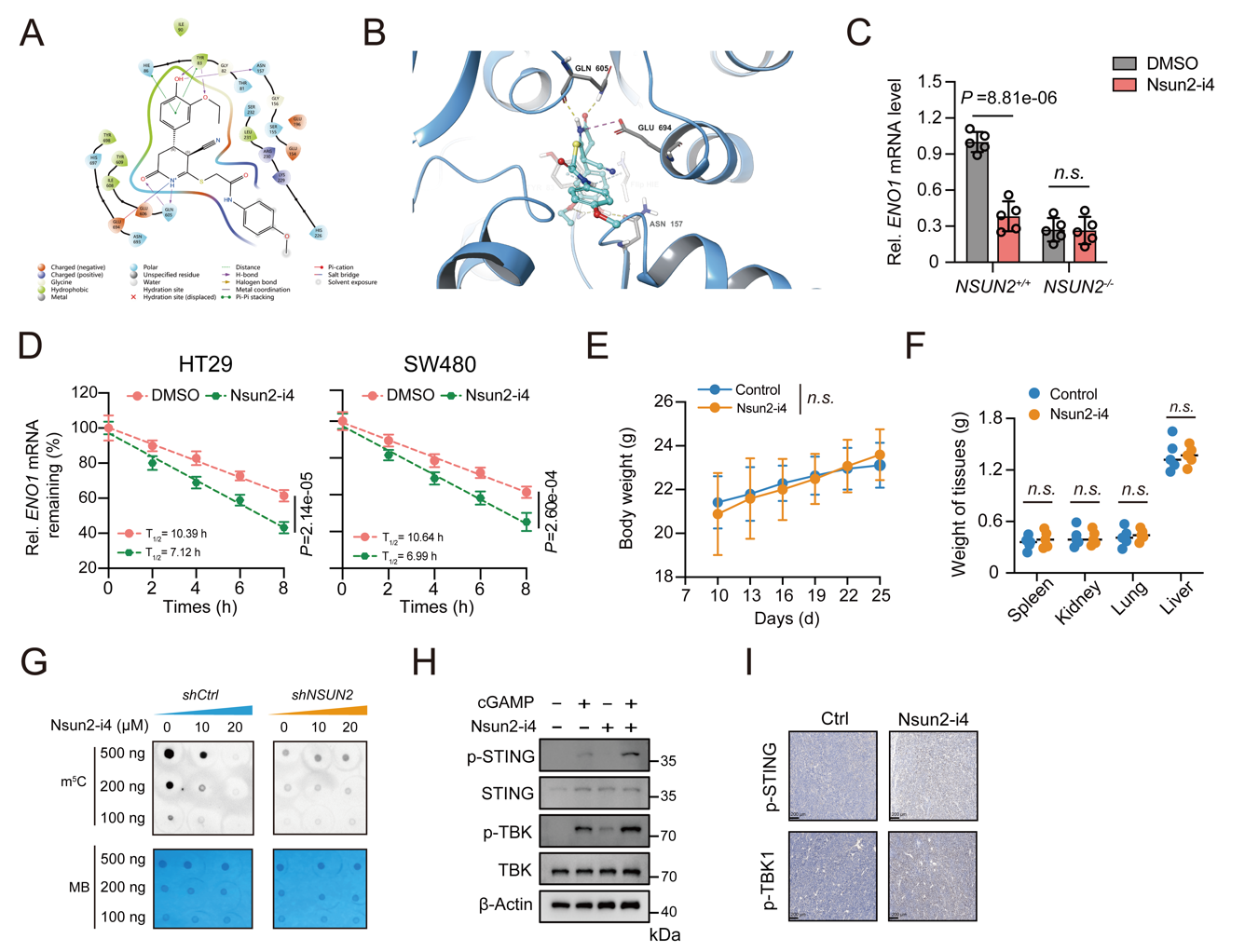
**

Figure S6. Identification of an Efficacious Small-molecule Inhibitor of NSUN2 in Combination with Immunotherapy. (A-B) The molecular docking of Nsun2-i4 and NSUN2 is shown based on the crystal structure of NSUN2. (C) RT-qPCR analysis of the relative *ENO1* mRNA expression level in WT or *NSUN2*-knockout CRC cells treated with DMSO or Nsun2-i4. (D) Effects of treatmen of Nsun2-i4 on mRNA half-life of ENO1 by RNA stability assays in CRC cells. (E) Body weight of animals in control and the Nsun2-i4 groups, at different time points. (F) Comparison of weights of organs and tissues between control and the Nsun2-i4 groups. (G) Effect of Nsun2-i4 on m^5^C abundance in mRNA transcriptomes of CRC cells by dot blot assay. (H) Immunoblotting analysis of influence of Nsun2-i4 on the cGAS-STING pathway. (I) Immunohistochemical analysis of Nsun2-i4 on the cGAS-STING pathway from animal assay.

Figure S7

**
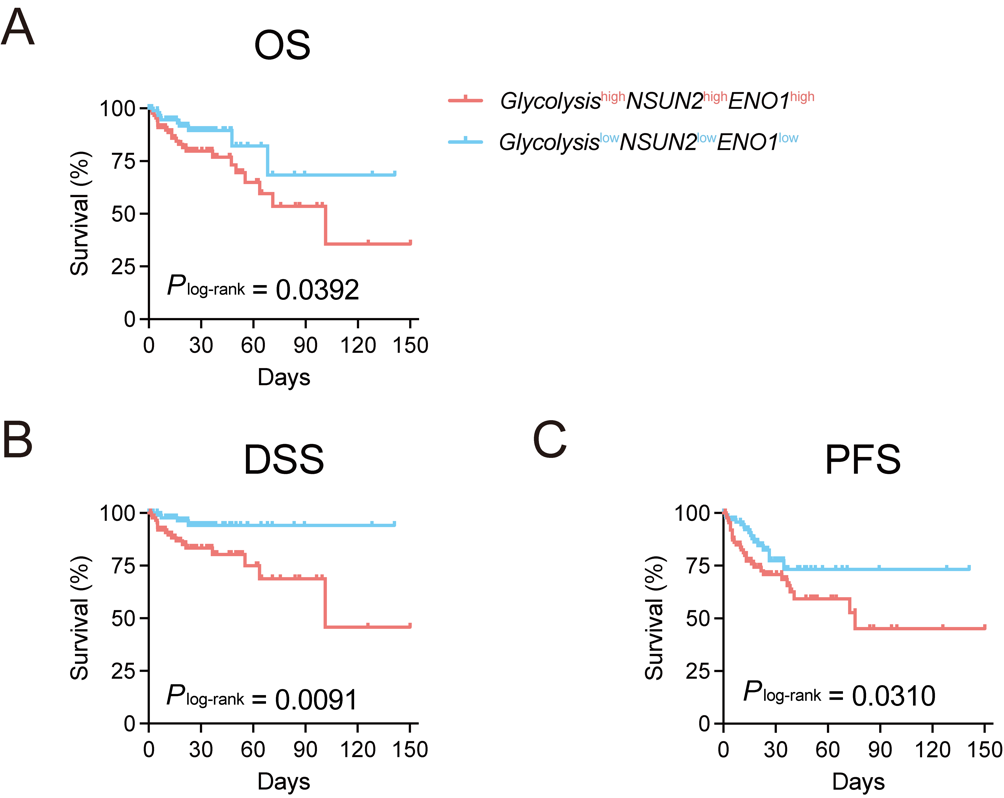
**

Figure S7. NSUN2 and ENO1 are correlated with glucose metabolism in CRC patients. (A) The survival curves of OS with high/low NSUN2, ENO1 and glycolysis signature in TCGA CRC cohorts. (B) The survival curves of DSS with high/low NSUN2, ENO1 and glycolysis signature in TCGA CRC cohorts. (C) The survival curves of PFS with high/low NSUN2, ENO1 and glycolysis signature in TCGA CRC cohorts.

Table S1. Clinicopathologic characteristics of patients enrolled for sample examination in 126 CRC.

| **No.** | **Age(y)** | **Gender** | **Tumor size** | **Tumor site** | **Grade** | **TNM** | **Blood vessel invasion** |
| --- | --- | --- | --- | --- | --- | --- | --- |
|  |  |  | **(cm)** |  |  |  |  |
| 1 | 61 | M | 6 | Colon | Low | T3N0M0 | Negative |
| 2 | 62 | F | 5 | Colon | Low | T3N1bM0 | Positive |
| 3 | 66 | F | 2.5 | Colon | Low | T3N2aM0 | Positive |
| 4 | 68 | M | 7 | Colon | Low | T4bN2bM0 | Positive |
| 5 | 66 | F | 2.9 | Colon | Low | T3N0M0 | Negative |
| 6 | 51 | M | 4 | Colon | Low | T3N0M0 | Negative |
| 7 | 64 | M | 5.5 | Colon | Low | T3N0M0 | Positive |
| 8 | 71 | F | 5 | Colon | High | T3N0M0 | Negative |
| 9 | 79 | M | 3.5 | Colon | Low | T3N0M0 | Negative |
| 10 | 77 | F | 4.5 | Rectum | Low | T3N1bM0 | Positive |
| 11 | 56 | M | 5 | Colon | Low | T3N0M0 | Negative |
| 12 | 58 | F | 5 | Colon | High | T4aN2aM0 | Positive |
| 13 | 68 | M | 8.5 | Colon | Low | T3N0M0 | Negative |
| 14 | 52 | F | 4 | Colon | Low | T2N1bM0 | Negative |
| 15 | 59 | F | 4 | Colon | Low | T3N0M0 | Positive |
| 16 | 79 | F | 3.5 | Colon | Low | T3N0M0 | Negative |
| 17 | 68 | M | 1.7 | Rectum | Low | T3N0M0 | Negative |
| 18 | 66 | M | 4 | Rectum | High | T3N2bM0 | Positive |
| 19 | 55 | F | 5 | Rectum | Low | T3N0M0 | Negative |
| 20 | 66 | M | 2 | Rectum | Low | T2N0M0 | Negative |
| 21 | 58 | M | 3 | Rectum | Low | T3N2aM0 | Positive |
| 22 | 57 | M | 5 | Rectum | Low | T3N2bM0 | Positive |
| 23 | 46 | M | 4 | Rectum | Low | T3N0M0 | Negative |
| 24 | 51 | M | 4 | Colon | High | T4N1aM0 | Positive |
| 25 | 40 | M | 4 | Colon | Low | T4N1cM0 | Positive |
| 26 | 87 | M | 4 | Rectum | Low | T3N2bM0 | Positive |
| 27 | 56 | F | 3 | Rectum | Low | T3N1bM0 | Positive |
| 28 | 60 | F | 8 | Colon | Low | T3N0M0 | Negative |
| 29 | 56 | F | 5 | Colon | High | T4aN1cM0 | Positive |
| 30 | 70 | M | 3 | Colon | High | T3N1M0 | Negative |
| 31 | 66 | M | 4 | Rectum | Low | T1N0M0 | Negative |
| 32 | 40 | M | 3.2 | Colon | Low | T3N0M0 | Negative |
| 33 | 53 | M | 3.5 | Rectum | High | T4N2M0 | Positive |
| 34 | 78 | F | 5 | Colon | Low | T2N0M0 | Negative |
| 35 | 66 | M | 7 | Colon | Low | T3N1M0 | Positive |
| 36 | 77 | M | 4.5 | Colon | High | T3N0M0 | Positive |
| 37 | 53 | F | 1.5 | Colon | Low | T2N0M0 | Negative |
| 38 | 84 | F | 4 | Rectum | Low | T3N2M0 | Positive |
| 39 | 65 | F | 5 | Rectum | Low | T3N1M0 | Positive |
| 40 | 21 | M | 3 | Rectum | Low | T2N0M0 | Negative |
| 41 | 58 | F | 7 | Colon | High | T3N1M0 | Negative |
| 42 | 58 | M | 4 | Rectum | Low | T2N0M0 | Negative |
| 43 | 52 | M | 5 | Rectum | High | T3N1M0 | Negative |
| 44 | 80 | M | 3 | Rectum | Low | T3N1M0 | Positive |
| 45 | 63 | F | 6 | Colon | Low | T4N1M0 | Positive |
| 46 | 54 | F | 3 | Rectum | Low | T3N0M0 | Negative |
| 47 | 48 | M | 6 | Colon | Low | T3N0M0 | Negative |
| 48 | 54 | F | 3 | Colon | Low | T3N2M0 | Negative |
| 49 | 71 | M | 3 | Rectum | Low | T1N1M0 | Negative |
| 50 | 60 | M | 6 | Rectum | Low | T2N0M0 | Negative |
| 51 | 71 | F | 4 | Colon | Low | T2N0M0 | Negative |
| 52 | 79 | M | 4 | Rectum | Low | T4N1M0 | Negative |
| 53 | 61 | M | 3 | Rectum | High | T2N2M0 | Positive |
| 54 | 64 | M | 3 | Colon | Low | T4N1M0 | Negative |
| 55 | 63 | F | 4 | Rectum | Low | T3N0M0 | Negative |
| 56 | 68 | F | 3.5 | Colon | Low | T3N1M0 | Positive |
| 57 | 56 | M | 4 | Rectum | Low | T3N1cM1 | Positive |
| 58 | 45 | M | 3.5 | Rectum | High | T3N2bM1 | Positive |
| 59 | 80 | F | 2.5 | Rectum | Low | T2N0M0 | Negative |
| 60 | 62 | F | 4.5 | Colon | Low | T3N1aM1 | Negative |
| 61 | 52 | F | 6 | Colon | Low | T3N0M0 | Negative |
| 62 | 64 | M | 3.5 | Colon | Low | T3N1aM0 | Positive |
| 63 | 78 | M | 8 | Colon | Low | T3N0M0 | Negative |
| 64 | 57 | F | 6 | Rectum | Low | T3N0M0 | Negative |
| 65 | 67 | M | 4 | Rectum | Low | T3bN1aM0 | Negative |
| 66 | 88 | M | 2.5 | Colon | Low | T3N0M0 | Negative |
| 67 | 47 | F | 3.5 | Rectum | Low | T3N1cM0 | Positive |
| 68 | 72 | M | 3 | Colon | Low | T3N1aM1 | Positive |
| 69 | 46 | M | 4 | Rectum | Low | T4aN2aM1c | Positive |
| 70 | 59 | M | 2.2 | Rectum | High | T2N0M0 | Negative |
| 71 | 61 | M | 4 | Rectum | Low | T2N0M0 | Negative |
| 72 | 70 | M | 1.5 | Rectum | Low | T2N0M0 | Negative |
| 73 | 62 | F | 6 | Colon | Low | T3N2bM1 | Positive |
| 74 | 70 | M | 5.5 | Rectum | High | T3N2M0 | Positive |
| 75 | 83 | F | 2 | Colon | Low | T2N0M0 | Negative |
| 76 | 49 | F | 3.5 | Colon | Low | T4bN1M1 | Negative |
| 77 | 75 | M | 1.6 | Rectum | Low | T2N0M0 | Positive |
| 78 | 63 | M | 3.5 | Colon | Low | T4N1M0 | Negative |
| 79 | 48 | M | 4.5 | Colon | Low | T3N0M0 | Negative |
| 80 | 58 | M | 5 | Rectum | Low | T3N1aM0 | Negative |
| 81 | 55 | F | 4.5 | Colon | Low | T4bN0M1 | Negative |
| 82 | 27 | M | 4.5 | Colon | High | T4aN1aM0 | Positive |
| 83 | 62 | M | 4 | Rectum | Low | T3N1cM0 | Negative |
| 84 | 59 | M | 4 | Rectum | Low | T3N1bM0 | Negative |
| 85 | 42 | M | 3.5 | Colon | Low | T4aN1M0 | Positive |
| 86 | 56 | F | 2.2 | Rectum | Low | T3N1M0 | Positive |
| 87 | 68 | M | 2.5 | Rectum | Low | T3N0M0 | Negative |
| 88 | 44 | F | 3.5 | Colon | Low | T3N1M0 | Positive |
| 89 | 46 | F | 8 | Colon | Low | T3N0M0 | Negative |
| 90 | 84 | M | 6.5 | Rectum | Low | T3N1cM0 | Negative |
| 91 | 79 | F | 2.5 | Colon | Low | T3N1M0 | Negative |
| 92 | 64 | M | 3.8 | Rectum | Low | T3N0M0 | Negative |
| 93 | 68 | M | 3.5 | Colon | Low | T3N0M1 | Negative |
| 94 | 83 | F | 7.5 | Colon | Low | T4bN0M0 | Negative |
| 95 | 26 | M | 6.5 | Colon | High | T3N0M0 | Negative |
| 96 | 57 | M | 2.2 | Colon | Low | T3N1bM0 | Negative |
| 97 | 62 | M | 3.5 | Rectum | Low | T3N0M0 | Negative |
| 98 | 68 | M | 5.5 | Rectum | Low | T3N1cM0 | Negative |
| 99 | 48 | F | 4.5 | Colon | Low | T2N0M0 | Negative |
| 100 | 62 | M | 5 | Colon | Low | T3N2M0 | Negative |
| 101 | 54 | M | 2 | Colon | High | T2N0M1 | Negative |
| 102 | 48 | M | 2.5 | Rectum | High | T2N0M0 | Negative |
| 103 | 87 | M | 2 | Colon | High | T4N1M0 | Negative |
| 104 | 50 | M | 5 | Colon | Low | T3N1M0 | Positive |
| 105 | 79 | M | 3.5 | Colon | High | T3N1M0 | Positive |
| 106 | 31 | M | 1.5 | Rectum | High | T3N0M0 | Negative |
| 107 | 52 | M | 3 | Rectum | High | T2N0M0 | Negative |
| 108 | 42 | M | 7.5 | Colon | High | T3N0M0 | Negative |
| 109 | 42 | F | 5.7 | Rectum | High | T2N0M0 | Negative |
| 110 | 46 | M | 6 | Colon | High | T3N0M0 | Positive |
| 111 | 78 | F | 5 | Colon | High | T4N1M0 | Positive |
| 112 | 55 | F | 4.5 | Rectum | High | T3N0M0 | Negative |
| 113 | 73 | F | 3.5 | Rectum | High | T2N0M0 | Negative |
| 114 | 39 | M | 10 | Colon | High | T3N0M0 | Positive |
| 115 | 63 | M | 4 | Colon | High | T3N0M0 | Negative |
| 116 | 66 | F | 2 | Rectum | High | T3N0M0 | Negative |
| 117 | 57 | F | 3.5 | Colon | Low | T3N1M1 | Positive |
| 118 | 80 | F | 4.5 | Rectum | High | T3N0M0 | Negative |
| 119 | 67 | M | 4 | Colon | High | T3N0M0 | Negative |
| 120 | 49 | M | 2 | Rectum | Low | T3N1M0 | Positive |
| 121 | 50 | M | 3.5 | Rectum | Low | T3N0M0 | Negative |
| 122 | 68 | F | 3.5 | Rectum | Low | T3N1M0 | Positive |
| 123 | 62 | M | 2.5 | Rectum | High | T2N0M0 | Negative |
| 124 | 59 | M | 4 | Rectum | High | T1N0M0 | Negative |
| 125 | 79 | M | 4 | Colon | High | T3N0M0 | Negative |
| 126 | 67 | F | 3 | Rectum | High | T2N0M0 | Positive |
|  |  |  |  |  |  |  |  |

Table S2. Primers for RT-qPCR.

| **Gene** |  | **Sequence** |
| --- | --- | --- |
| NOP2 | Forward | 5′- GGGCACAGACACACAAAC -3′′ |
|  | Reverse | 5′- GAACGGATGGGAGACACA G -3′ |
| NSUN2 | Forward | 5′- GGTATCCTGAAGAACTTGCC -3′ |
|  | Reverse | 5′- ATCTTATGATGAGGCCGCA -3′ |
| NSUN3 | Forward | 5′- CACGCTTTCCAAGGCAGAAA -3′  CACGCTTTCCAAGGCAGAAA  CACGCTTTCCAAGGCAGAAA  - 3′ |
|  | Reverse | 5′-TGAAGTCGTGGGAGCAAGTC -3′  TGAAGTCGTGGGAGCAAGTC  TGAAGTCGTGGGAGCAAGTC  -3′ |
| NSUN4 | Forward | 5′-CCAAACCCTGGCAAAAGGTG -3′ |
|  | Reverse | 5′-GCGTGCCGGTCATAGAAGAA -3′  GCGTGCCGGTCATAGAAGAA  GCGTGCCGGTCATAGAAGAA  GCGTGCCGGTCATAGAAGAA  GCGTGCCGGTCATAGAAGAA |
| NSUN5 | Forward | 5′- CCTCGATGACTTACGAGCCC -3′ |
|  | Reverse | 5′- TGATGCTTTGGCCTGTGAGG -3′ |
| NSUN6 | Forward | 5′- CAGAATGCCTTATTGTTAGGGCT -3′ |
|  | Reverse | 5′- ACCATATCAAGTTTAACCGCCTT -3′ |
| NSUN7 | Forward | 5′-GAGGCTGAGCTTTCCTCGG -3′ |
|  | Reverse | 5′- AGGCCGTTTGGTTCAAATGC -3′ |
| TRDMT1 | Forward | 5′- TGCCAGCCATTCACAAGGAT -3′ |
|  | Reverse | 5′- ACACAGACCCTGTCCCTTCT -3′ |
| DNMT1 | Forward | 5′- GAGGAGGGCTACCTGGCTAA -3′ |
|  | Reverse | 5′- GCTTAGCCTCTCCATCGGAC -3′ |
| DNMT3A | Forward | 5′- TTCACCAGAGGGCTCAACAC -3′ |
|  | Reverse | 5′- CGGGAGCCCTCCATTTTCAT -3′ |
| DNMT3B | Forward | 5′- CCGCTTCCTCGCAGCAG -3′ |
|  | Reverse | 5′- TGGGCTTTCTGAACGAGTCC -3′ |
| ALYREF | Forward | 5′- GCAGGCCAAAACAACTTCCC -3′ |
|  | Reverse | 5′- CTCAAAGTGCACGTCTGCTG -3′ |
| YTHDF2 | Forward | 5′- CGAGTGTCAGGGACAAAAGC -3′ |
|  | Reverse | 5′- CCGTAGACCAAGCAGCTTCA -3′ |
| YBX1 | Forward | 5′- GAGAAGTGATGGAGGGTGCT -3′ |
|  | Reverse | 5′- TTAGGGTTTTCTGGGCGTCT -3′ |
| TET1 | Forward | 5′- ACTCCCTGAGGTCTGTCCTG -3′ |
|  | Reverse | 5′- CACAAGGTTTTGGTCGCTGG -3′ |
| TET2 | Forward | 5′- GCAGCACACCCTCTCAAGAT -3′ |
|  | Reverse | 5′- TGGTTTTCTGCACCGCAATG -3′ |
| TET3 | Forward | 5′- AGCGCTAAAGCAAGGAAAGA -3′ |
|  | Reverse | 5′- CAGGGGAGGAAGGAGATCCA -3′ |
| ALKBH1 | Forward | 5′- ACAGTCCGCCATCTTTCTCC -3′ |
|  | Reverse | 5′- AATTCTGGTCTGTGGCCAGG -3′ |
| ENO1 | Forward | 5′- CGGCTTTACGTTCACCTCGG -3′ |
|  | Reverse | 5′- TCAACAGCCTTTGAGACACCCT -3′ |
| GAPDH | Forward | 5′- TGCACCACCAACTGCTTAGC -3′ |
|  | Reverse | 5′- GGCATGGACTGTGGTCATGAG -3′ |

Table S3. Mouse genotypes primers.

| **Gene Name** |  | **Primers** |
| --- | --- | --- |
| Mouse-NSUN2-Left-1 | Forward | 5′-CAAACTCAGAGAGACAATCCCCTC- 3′ |
| Mouse-NSUN2-Right-1 | Reverse | 5′-CCTCTAATAGTCACCTTCCCTCAC- 3′ |
| Mouse-NSUN2-Left-2 | Forward | 5′-CAAACTCAGAGAGACAATCCCCTC- 3′ |
| Mouse-NSUN2-Right-2 | Reverse | 5′-ATTAATCTCTGTGTTGGCACTGAC-3′ |

Table S4: Specific primers used for Bisulfite PCR Pyrosequencing of *ENO1* RNA.

| Gene |  | Sequence |
| --- | --- | --- |
| ENO1 | Forward | 5′- AGTATTTGTGGGTATTTGGA - 3′′ |
| ENO1 | Reverse | 5′- GGAATTTTATTGTTGAGGTT - 3′ |

Table S5. Targeted sequences of shRNAs or siRNAs and probe sequences of ENO1 used in this study.

| shRNA | Sequence |
| --- | --- |
| sgNSUN2 Top CACCAGATCGTGCCCGAGGGCGAG 3’ | 5'- CACCAGATCGTGCCCGAGGGCGAG -3' |
| sgNSUN2 Bottom | 5'- AAACCTCGCCCTCGGGCACGATCT -3' |
| shNSUN2 | 5'- CACGTGTTCACTAAACCCTAT -3'  -3'  -3' |
| shENO1#1 | 5'- GGACTTTCAAGTCTCCCGATGA -3' |
| shENO1#2 | 5'- GCTCAAAGTCAACCAGATTG -3' |
| shYBX1#1 | 5'- GAGAACCCTAAACCACAAGAT -3' |
| shYBX1#2 | 5'- GTATCGCCGAAACTTCAATTA -3' |
| shControl | 5'- ATCTCGCTTGGGCGAGAGTAAG -3'  -3' |
| siRNA-LDHA | 5′- GGCAAAGACUAUAAUGUAA -3′ |
| siRNA-LDHB | 5′- GGCAACAGTTCCAAACAATAAGA -3′ |
| Probe sequences for RNA pulldown | |
| ENO1 [C] | GATCTCGCCGGCTTTACGTTCACCTCGGTGTCTGCAGCACCCTCCGCTTC-Biotin |
| ENO1 [m^5^C] | GATCTCGCCGGCTTTACGTTCACCT[m^5^C]GGTGTCTGCAGCACCCTCCGCTTC-Biotin |

Table S6. Clinicopathologic characteristics of CRC patients with PET-CT.

| No. | Age(y) | Gender | Tumor size | Tumor site | Grade | TNM | Blood vessel invasion |
| --- | --- | --- | --- | --- | --- | --- | --- |
|  |  |  | (cm) |  |  |  |  |
| 1 | 54 | M | 2 | Colon | High | T2N0M1 | Negative |
| 2 | 48 | M | 2.5 | Rectum | High | T2N0M0 | Negative |
| 3 | 87 | M | 2 | Colon | High | T4N1M0 | Negative |
| 4 | 50 | M | 5 | Colon | Low | T3N1M0 | Positive |
| 5 | 79 | M | 3.5 | Colon | High | T3N1M0 | Positive |
| 6 | 31 | M | 1.5 | Rectum | High | T3N0M0 | Negative |
| 7 | 52 | M | 3 | Rectum | High | T2N0M0 | Negative |
| 8 | 42 | M | 7.5 | Colon | High | T3N0M0 | Negative |
| 9 | 42 | F | 5.7 | Rectum | High | T2N0M0 | Negative |
| 10 | 46 | M | 6 | Colon | High | T3N0M0 | Positive |
| 11 | 78 | F | 5 | Colon | High | T4N1M0 | Positive |
| 12 | 55 | F | 4.5 | Rectum | High | T3N0M0 | Negative |
| 13 | 73 | F | 3.5 | Rectum | High | T2N0M0 | Negative |
| 14 | 39 | M | 10 | Colon | High | T3N0M0 | Positive |
| 15 | 63 | M | 4 | Colon | High | T3N0M0 | Negative |
| 16 | 66 | F | 2 | Rectum | High | T3N0M0 | Negative |
| 17 | 57 | F | 3.5 | Colon | Low | T3N1M1 | Positive |
| 18 | 80 | F | 4.5 | Rectum | High | T3N0M0 | Negative |
| 19 | 67 | M | 4 | Colon | High | T3N0M0 | Negative |
| 20 | 49 | M | 2 | Rectum | Low | T3N1M0 | Positive |
| 21 | 50 | M | 3.5 | Rectum | Low | T3N0M0 | Negative |
| 22 | 68 | F | 3.5 | Rectum | Low | T3N1M0 | Positive |
| 23 | 62 | M | 2.5 | Rectum | High | T2N0M0 | Negative |
| 24 | 59 | M | 4 | Rectum | High | T1N0M0 | Negative |
| 25 | 79 | M | 4 | Colon | High | T3N0M0 | Negative |
| 26 | 67 | F | 3 | Rectum | High | T2N0M0 | Positive |

Table S7. The antibodies used in this study.

| Antibody | Supplier | Catalogue | | Host |  |  |
| --- | --- | --- | --- | --- | --- | --- |
| Anti-NSUN2 | Proteintech | 20854-1-AP | | Rabbit |  |  |
| Anti-ENO1 | Abclonal | A1033 | | Rabbit |  |  |
| Anti-YBX1 | Proteintech | 20339-1-AP | | Rabbit |  |  |
| Anti-phospho-STING | Cell Signaling Technology | 50907 | | Rabbit |  |  |
| Anti-phospho-TBK1 | Cell Signaling Technology | 5483 | | Rabbit |  |  |
| Anti-Flag | Proteintech | 66008-4-Ig | | Mouse |  |  |
| Anti-HA | | Proteintech | 51064-2-AP | Rabbit | | |
| Anti-GRB2 | Abclonal | A19059 | | Rabbit |  |  |
| Anti-H3K18 la | PTM Bio | PTM-1406RM | | Rabbit |  |  |
| Anti-H3K23 la | PTM Bio | PTM-1413 | | Rabbit |  |  |
| Anti-H4K8 la | PTM Bio | PTM-1405 | | Rabbit |  |  |
| Anti-H4K12 la | PTM Bio | PTM-1411 | | Rabbit |  |  |
| Anti-L-Lactyl Lysine | PTM Bio | PTM-1401RM | | Rabbit |  |  |
| Anti-H3 | Proteintech | 17168-1-AP | | Rabbit |  |  |
| Anti-GAPDH | Proteintech | 60004-1-Ig | | Mouse |  |  |
| Anti-β-Actin | Abclonal | AC004 | | Mouse |  |  |
| Anti-Lamin B1 | Proteintech | 12987-1-AP | | Mouse |  |  |
| HRP Goat Anti-Mouse IgG (H+L) | Abclonal | AS003 | | Mouse |  |  |
| HRP Goat Anti-Rabbit IgG (H+L) | Abclonal | AS014 | | Rabbit |  |  |
| Fluorescein (FITC)–conjugated Affinipure Goat Anti-Mouse IgG (H+L) | Proteintech | SA00003-1 | | Mouse |  |  |
| Cy3–conjugated Affinipure Goat Anti-Rabbit IgG (H+L) | Proteintech | SA00009-2 | | Rabbit |  |  |

Table S8. The small-molecule compounds or drugs used in our study.

| Name | Supplier | Catalogue |
| --- | --- | --- |
| Nsun2-i1 | [ScreeningCompound](https://www.screeningcompound.com/pages/437.html) | HIT211724083 |
| Nsun2-i2 | [ScreeningCompound](https://www.screeningcompound.com/pages/437.html) | HIT214960013 |
| Nsun2-i3 | [ScreeningCompound](https://www.screeningcompound.com/pages/437.html) | HIT101726260 |
| Nsun2-i4 | [ScreeningCompound](https://www.screeningcompound.com/pages/437.html) | HIT101586182 |
| Nsun2-i5 | [ScreeningCompound](https://www.screeningcompound.com/pages/437.html) | HIT101540326 |
| AOM | Sigma-Aldrich | A5486 |
| DSS | MP Biomedicals | 02160110-CF |
| Actinomycin D | Sigma-Aldrich | A9415 |
| L- (+) -Lactic acid | Sigma-Aldrich | L-6402 |
| Sodium L-lactate | Sigma-Aldrich | 71718 |
| Rotenone | Sigma-Aldrich | R8875 |
| C646 | MedChemExpress | HY-13823 |
| A485 | MedChemExpress | HY-107455 |
|  |  |  |
